# Candidate genes for limiting cholestatic intestinal injury identified by gene expression profiling

**DOI:** 10.1002/phy2.73

**Published:** 2013-09-17

**Authors:** Samuel M Alaish, Jennifer Timmons, Alexis Smith, Marguerite S Buzza, Ebony Murphy, Aiping Zhao, Yezhou Sun, Douglas J Turner, Terez Shea-Donahue, Toni M Antalis, Alan Cross, Susan G Dorsey

**Affiliations:** 1 Department of Surgery, University of Maryland School of MedicineBaltimore, Maryland; 2 Department of Physiology, University of Maryland School of MedicineBaltimore, Maryland; 3 Department of Medicine, University of Maryland School of MedicineBaltimore, Maryland; 4 Institute for Genome Sciences, University of Maryland School of MedicineBaltimore, Maryland; 5 Baltimore Veterans Administration Medical CenterBaltimore, Maryland; 6 University of Maryland School of NursingBaltimore, Maryland

**Keywords:** Cholestasis, growth hormone, intestine, lipocalin, microarray

## Abstract

The lack of bile flow from the liver into the intestine can have devastating complications including hepatic failure, sepsis, and even death. This pathologic condition known as cholestasis can result from etiologies as diverse as total parenteral nutrition (TPN), hepatitis, and pancreatic cancer. The intestinal injury associated with cholestasis has been shown to result in decreased intestinal resistance, increased bacterial translocation, and increased endotoxemia. Anecdotal clinical evidence suggests a genetic predisposition to exaggerated injury. Recent animal research on two different strains of inbred mice demonstrating different rates of bacterial translocation with different mortality rates supports this premise. In this study, a microarray analysis of intestinal tissue following common bile duct ligation (CBDL) performed under general anesthesia on these same two strains of inbred mice was done with the goal of identifying the potential molecular mechanistic pathways responsible. Over 500 genes were increased more than 2.0-fold following CBDL. The most promising candidate genes included major urinary proteins (MUPs), serine protease-1-inhibitor (Serpina1a), and lipocalin-2 (LCN-2). Quantitative polymerase chain reaction (qPCR) validated the microarray results for these candidate genes. In an in vitro experiment using differentiated intestinal epithelial cells, inhibition of MUP-1 by siRNA resulted in increased intestinal epithelial cell permeability. Diverse novel mechanisms involving the growth hormone pathway, the acute phase response, and the innate immune response are thus potential avenues for limiting cholestatic intestinal injury. Changes in gene expression were at times found to be not only due to the CBDL but also due to the murine strain. Should further studies in cholestatic patients demonstrate interindividual variability similar to what we have shown in mice, then a “personalized medicine” approach to cholestatic patients may become possible.

## Introduction

Cholestasis, defined as little or no bile flow from the liver into the intestine, is a complex pathologic condition that can develop from either functional etiologies, such as hepatic parenchymal disease secondary to hepatitis, or mechanical etiologies, such as an obstructing pancreatic cancer or biliary stricture. In the pediatric population, cholestasis resulting from prolonged parenteral nutrition is by far the most common etiology. Cholestatic injury has not only a hepatic component but also an intestinal one. Failure of the intestinal barrier with decreased intestinal resistance, increased bacterial translocation, and increased episodes of sepsis has been well described (Campillo et al. 1999; Pascual et al. 2003; Frances et al. 2004); however, the exact mechanisms remain poorly understood.

Common bile duct ligation (CBDL) is a standard model of cholestasis in the literature (Georgiev et al. 2008). CBDL in mice leads to both hepatic and intestinal injuries which are precisely interrelated. We have previously found differences in the systemic inflammatory responses and outcome following CBDL between two inbred mouse strains, C57BL/6J (B6) and A/J, suggesting a genetic contribution (Alaish et al. 2005). In particular, B6 mice were significantly more likely to develop ascites following 1 week of CBDL (Alaish et al. 2005). In concordance with this observation, the frequency of mortality after CBDL was significantly higher in B6 mice compared to A/J mice on days following CBDL (Alaish et al. 2005). Interestingly, although both strains demonstrated markedly elevated plasma liver function tests following CBDL, no difference was noted in liver histology between the two ligated strains. More recently, our laboratory has shown decreased intestinal resistance and increased bacterial translocation following CBDL in these same two strains of inbred mice. Furthermore, we found genetic variation in the intestinal resistance and bacterial translocation rates, which correlated with mortality following CBDL in different strains of inbred mice (Alaish et al. 2013). Further analysis implicated an IFN-γ-mediated apoptotic-independent mechanism of tight junction disruption, which has been well described in vitro (Madara and Stafford 1989; Marano et al. 1998; Youakim and Ahdieh 1999; Bruewer et al. 2003; Clayburgh et al. 2004), as a mechanism possibly responsible for the genetic variation. Nevertheless, the 2.5-fold changes in IFN-γ gene expression following CBDL, albeit significant, did not seem monumental enough to fully explain the striking genetic influence on mortality following CBDL in the mice. In order to uncover other potential mechanisms including novel pathways, we embarked on a whole-genome microarray analysis of jejunal tissue in these two different strains of inbred mice following either a sham operation or CBDL. The differentially expressed genes reported here constitute a resource of candidate genes for roles in cholestatic intestinal injury.

## Material and Methods

### Animals

Male A/J and C57BL/6J (B6) mice (8 weeks old) were obtained from the Jackson Laboratory (Bar Harbor, ME) and maintained in identical environmental conditions in a pathogen-free animal facility with 12-h light–dark cycles. All mice weighed 18–25 g at the time of operation. Matriptase (*St14*) hypomorphic C57BL/6J mice (List et al. 2007) were bred in the Antalis laboratory. Animal studies were conducted according to protocols reviewed and approved by the University of Maryland School of Medicine Institutional Animal Care and Use Committee and adhered to guidelines promulgated by the National Institutes of Health. In accordance with these guidelines, we used the minimum number of animals to meet the rigor necessary for this series of experiments.

### Experimental design

#### CBDL operative procedure

Mice were anesthetized by inhaled isoflurane anesthesia. The abdomen was clipped and then prepared in sterile fashion with 70% ethyl-ethanol followed by betadine. A transverse upper abdominal incision was performed. The CBD was dissected away from the portal vein and was ligated near its junction with the duodenum using aneurysm clips engineered with a precisely standardized opening/closing mechanism. The abdominal wall was then closed in a two-layer fashion using absorbable sutures. Sham-operated mice were treated identically but without dissection or ligation of the CBD. Postoperatively, animals were resuscitated with warmed subcutaneous injections of saline (1 mL) to replace losses. Mice were returned to clean cages where food and water were provided ad libitum. Buprenorphine, 0.05–0.1 mg/kg was given subcutaneously at the time of surgery and then every 8–12 h to treat postoperative pain for 48–72 h.

#### RNA extraction

Seven days following the surgery, the mice underwent deep general anesthesia and euthanasia by thoracotomy and cardiac exsanguination. Postoperative day 7 was chosen because this time point corresponded to our earlier finding of decreased intestinal transepithelial electrical resistance (TEER) after CBDL (Alaish et al. 2013). In addition, this time point exhibited differences in TEER based on the genetic background of the mouse (Alaish et al. 2013). These TEER findings were found in both the jejunum and ileum and correlated with differences in bacterial translocation and mortality. Further studies on jejunal tissue demonstrated differences in tight junction protein expression between CBDL and sham animals and between the strains (Alaish et al. 2013). Therefore, in this study, we chose jejunum once again; the intestinal tissue was harvested under sterile conditions. RNA extraction and purification were performed as we have previously described (Dorsey et al. 2009).

#### Microarray data analysis

Microarray expression profiling was performed according to the manufacturer protocols (Affymetrix, Santa Clara, CA). Briefly, total RNA was used to prepare biotinylated cRNA, followed by fragmentation and hybridization to Affymetrix arrays (Genechip Mouse 430 2.0; Affymetrix, Santa Clara, CA). The arrays were incubated for approximately 16 h, washed, stained, and scanned per Affymetrix. Differential gene expression through microarray was then performed. We utilized.cel files generated from Affymetrix profiling process for analysis. Arrays were normalized by GCRMA method implemented in gcrma R package (Bioconductor, an open source collection of software packages). Differential expression analysis was performed using limma R package (Bioconductor). First, a linear model was fitted to expression data for each gene. Empirical Bayes method was then used to assess differential expression between two conditions. A cutoff of FDR less than 0.05 was used to select significant probes. A complete data set has been submitted to the NCBI Gene Expression Omnibus (NCBI GEO #GSE47099 and NCBI Tracking System #16793295).

#### qPCR verification of promising candidate genes

The identification of significant changes in expression of promising candidate genes (major urinary proteins [MUPs], serine protease-1-inhibitor [Serpina1a] and lipocalin-2 [LCN2]) through microarray analysis was validated using a quantitative polymerase chain reaction (qPCR) technique. Total RNA was isolated from homogenized jejunal samples that were stored in TRIzol (Invitrogen, Grand Island, NY). The total RNA was isolated from TRIzol samples as per the manufacturer's instructions. The pellet was allowed to air dry, and the total RNA was resuspended in an appropriate volume of RNAse-free water. RNA concentrations were calculated using a NanoDrop 1000 spectrophotometer (Thermo Scientific, Waltham, MA). Single-stranded cDNA was synthesized from 2 μg of total RNA using random hexamer primer and the First-Strand cDNA Synthesis Kit (MBI Fermentas, Hanover, MD). The specific primer sequences were designed using Beacon Designer 7.0 (Premier Biosoft International, Palo Alto, CA) and synthesized by the University of Maryland School of Medicine Biopolymer/Genomics Core. qPCR reactions were set up using iQ SYBR Green Supermix (Bio-Rad, Hercules, CA) in a total volume of 25 μL. Amplification conditions were as follows: 95°C for 3 min, 50 cycles of 95°C for 15 sec, 60°C for 15 sec, and 72°C for 20 sec. All reactions were performed using Bio-Rad iCycler instrumentation and software. All samples were normalized with 18s rRNA housekeeping gene levels with subsequent calculation of fold change in mRNA expression. Analysis was carried out in GraphPad Prism5 (San Diego, CA, USA).

#### Mortality following CBDL in matriptase hypomorphic B6 mice

We conducted a mortality study following CBDL in wild-type C57BL/6J mice (*n* = 8) and matriptase (*St14*) hypomorphic C57BL/6J mice (*n* = 11). Sham-operated mice of each strain served as controls.

#### Cells

Cdx2-intestinal epitheial cells (Cdx2-IEC), a transformed rat crypt IEC-6 cell line which maintains a stable differentiated phenotype upon future passages, were obtained from Dr. J.-Y. Wang (University of Maryland, Baltimore, MD). Cdx2-IEC cells were maintained at 37°C in a humidified incubator with 10% CO_2_ in DMEM containing 5% (v:v) fetal bovine serum (FBS), 0.5% (v:v) ITS + liquid media supplement, 0.1 million units/L penicillin, 100 mg/L streptomycin, and 4 mmol/L sopropylthio-β-d-galactoside, which served as an inducer.

#### Transfection of Cdx2-IEC cells with MUP-1 siRNA and FITC-dextran permeability assay

Following their sixth passage, Cdx2-IEC cells were transfected with either MUP-1 siRNA (Thermo Scientific, Inc.) or Acell Control Non-Targeting siRNA (Thermo Scientific, Inc.) as described previously (Rao et al. 2006). The siRNAs used were as follows: 80 nmol/L Control siRNA; and 20 nmol/L, 40 nmol/L, and 60 nmol/L MUP-1 siRNA. Silencing of MUP-1 in the cells was confirmed by Western blot analysis using MUP (F-3) mouse monoclonal antibody (Santa Cruz Biotechnology, Inc., Paso Robles, CA, USA). Six-well transwell plates with 12-mm-diameter inserts (Costar 3407; Corning, Inc., Kennebunk, ME, USA) were used to perform the permeability studies following the transfection. The cells were incubated on the inserts with control media for 24 h to allow proper attachment to the membrane prior to dextran administration. After 24 h, TEER was measured in both control and transfected Cdx2-IEC cells for the formation of the monolayer as described previously (El Asmar et al. 2002). The media were removed. 4-kDa FITC-dextran in control media was placed onto the apical side (top chamber); control media alone was placed on the basolateral side. The TEER was monitored for 2 h and 100-μL aliquots of the basolateral medium were collected after each 30-min time period. A sample from the top compartment at the time of the last sampling of the bottom compartment was used to normalize the samples to account for possible differences in the total fluorescence added at the beginning of the experiment. Fluorescence of the samples was quantified in a multiplate fluorescence reader in black 96-well plates; the excitation wavelength was 485 nm and emission wavelength was 538 nm.

### Statistical analysis

The microarray data (reported as percent change) were analyzed using repeated measures analyses of variance (ANOVA) with false discovery rate correction to control multiple testing errors. Post hoc testing was done using Tukey's honestly significant difference (HSD). qPCR data were analyzed using ANOVA with the Bonferroni posttest. Graph Pad Prism 5 software was used. For the matriptase hypomorphic B6 mouse CBDL experiment, a Kaplan–Meier survival curve was generated with *P* < 0.05 considered as significant.

## Results

### Microarray data

**Figure 1:**
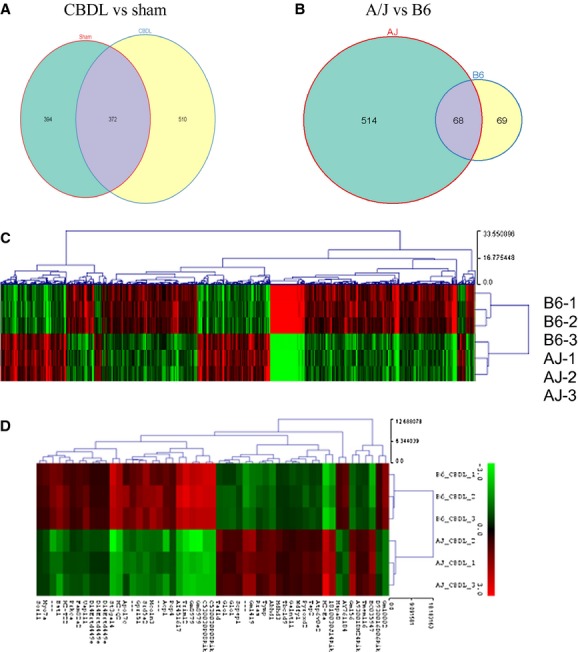
Venn diagram and heat map depicting the number of statistically significant differentially expressed genes by experimental condition and across murine strains. The venn diagram in (A) depicts the number of genes that are differentially expressed in the surgical group versus sham. In (B) we show the number of differentially expressed genes across two strains, A/J and B6. (C) The heat map shows all differentially expressed genes between strains. (D) A heat map which shows the top 53 significantly regulated genes across two strains. For both heat maps, red = upregulated genes; green = downregulated genes.

As shown in Figure 1A, more than 500 genes were significantly differentially regulated in the CBDL mice compared with sham. When examining changes across strains, although there are shared genes, there are a significant number of differentially regulated genes that are unique to each strain (Fig. 1B). Table 1 shows the number of genes in each category**.** The first two lines of data in Table 1 demonstrate that many more genes are differentially expressed in A/J mice as compared to B6 mice following CBDL (582 vs. 137). The last two lines of data in Table 1 demonstrate that there are more genes undergoing expression changes following CBDL compared to sham (882 vs. 766). In Figure 1C, the heat map shows clustering of all differentially expressed genes by genotype. A list of all differentially expressed genes can be found in Table A1. In Figure 1D, the top 53 significantly regulated genes across the two strains are shown in a separate heat map (For both heat maps, red = upregulated genes; green = downregulated genes). Note the disparate gene expressions in the two strains.

**Table 1:** Differentially expressed genes with FDR < 0.05

	Total	Up	Down
A/J CBDL versus sham	582	404	178
B6 CBDL versus sham	137	76	61
Sham A/J versus B6	766	276	490
CBDL A/J versus B6	882	291	591

**Figure 2:**
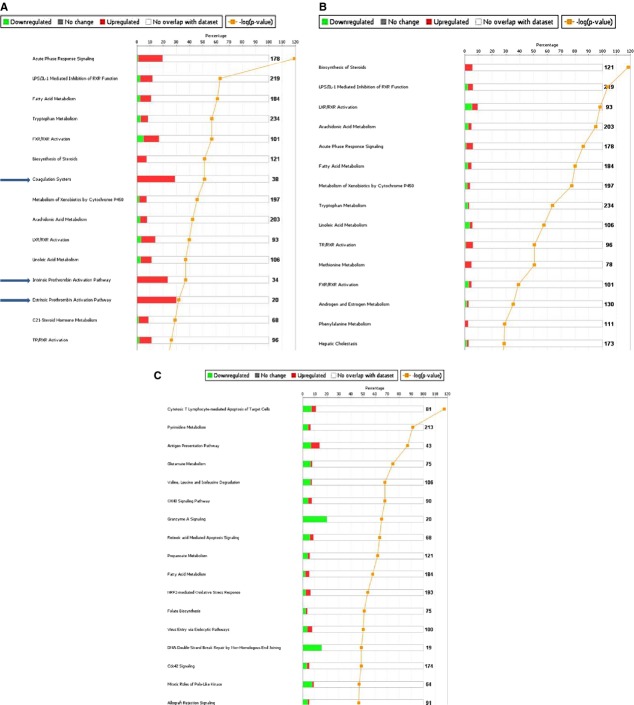
Significantly enriched canonical signaling pathways from differentially expressed gene sets. As denoted by the blue arrows in (A), coagulation pathways were highly upregulated in CBDL AJ mice compared with sham. In contrast, coagulation pathways were not significantly regulated in B6 CBDL versus sham (B). In (C), we show that there are a number of signaling pathways that are differentially regulated in AJ CBDL compared with B6 CBDL, demonstrating differential pathway activation in CBDL across these two inbred strains of mice.

We next examined significantly enriched canonical signaling pathways. First, we evaluated those that were unique within a strain in the CBDL condition compared with sham. As shown in Figure 2A, coagulation pathways were highly upregulated in A/J CBDL mice compared with sham. In contrast, coagulation pathways were not significantly regulated in B6 CBDL versus sham (Fig. 2B). In Figure 2C, we show that there are a number of signaling pathways that are differentially regulated in A/J CBDL compared with B6 CBDL, demonstrating differential pathway activation in CBDL across these two inbred strains of mice.

### qPCR verifies microarray results of candidate genes

**Figure 3:**
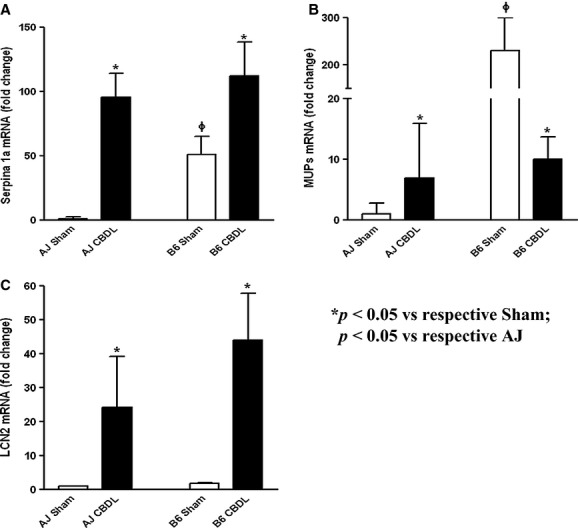
Serpina 1a, MUPs, and LCN2 gene expression changes determined by qPCR are consistent with microarray findings. (A) demonstrates a marked increase in Serpina 1a mRNA following CBDL in both strains. Also, there is a strain difference with increased Serpina 1a mRNA expression in B6 as compared to A/J mice. (B) demonstrates marked strain differences in MUP mRNA expression in the sham animals with disparate responses to CBDL. A/J sham MUP mRNA expression is low and expression increases following CBDL; whereas, in the B6 strain, MUP mRNA expression is very high in the sham group but falls in the CBDL group. (C) shows low expression of LCN2 mRNA in sham animals with marked increases following CBDL in both strains.

The following genes struck us as particularly promising as candidate genes to limit cholestatic injury: MUPs, Serpina1a, and LCN2. They all had significant changes in gene expression following CBDL. MUPs and Serpina1a also had disparate strain expressions which could account for the phenotypic differences we see following ligation. LCN2 was chosen because it is known to play a protective role in the intestinal barrier (Berger et al. 2006). Although we did not see strain differences in LCN2, we did find striking differences between the sham and CBDL mice, which we believe is an important finding. This study not only illustrates strain differences following CBDL but also serves to illustrate intestinal gene expression changes following CBDL independent of strain. qPCR did, indeed, verify our results for MUPs, Serpina1a, and LCN2. (Fig. 3A–C).

### Matriptase (St14) hypomorphic B6 mice do not have significantly increased mortality following CBDL compared to wild-type B6 mice

**Figure 4:**
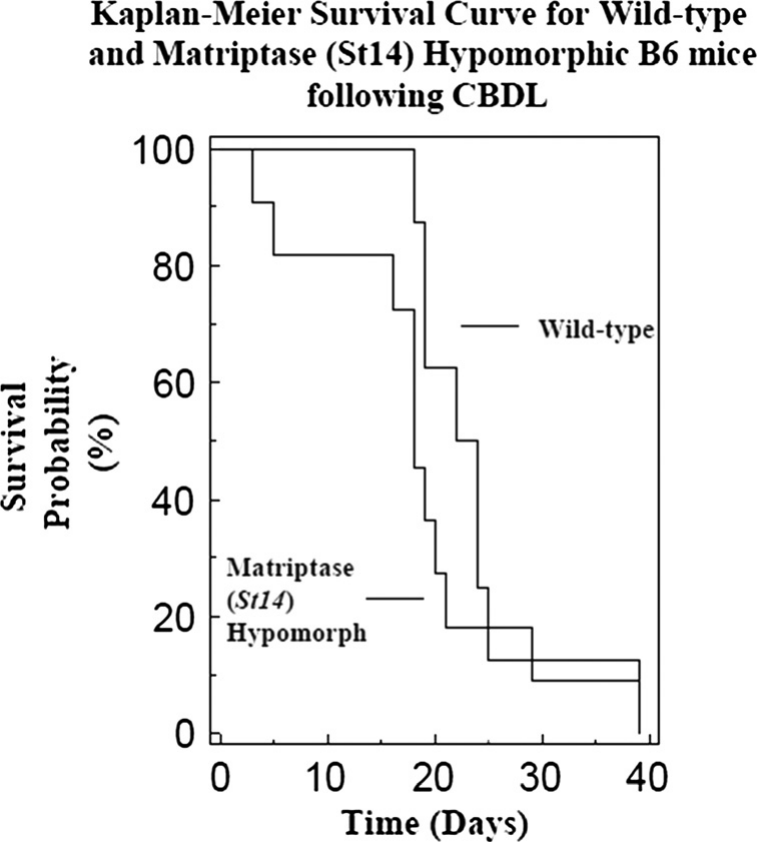
Kaplan–Meier survival curve for matriptase hypomorphic B6 and wild-type B6 mice following CBDL. The early trend toward increased mortality in the matriptase hypomorphic B6 mice is not statistically significant.

Serpina1a is a serine protease inhibitor, suggesting that decreased intestinal serine protease activity could contribute to the decreased intestinal resistance associated with CBDL. Matriptase is a serine protease whose loss results in decreased intestinal resistance as measured by decreased intestinal TEER (Buzza et al. 2010). We hypothesized that when coupled with the loss of matriptase, the increase in Serpina1a which follows CBDL would result in such a large drop in intestinal resistance that mortality would increase. Although there appeared to be a trend toward increased mortality in the matriptase (*St14*) hypomorphic B6 mice early on following CBDL, this difference never became significant and did not persist, as can be seen in the Kaplan–Meier Survival Curve (Fig. 4).

### MUP-1 siRNA transfection increases permeability in Cdx2-IEC cells

**Figure 5:**
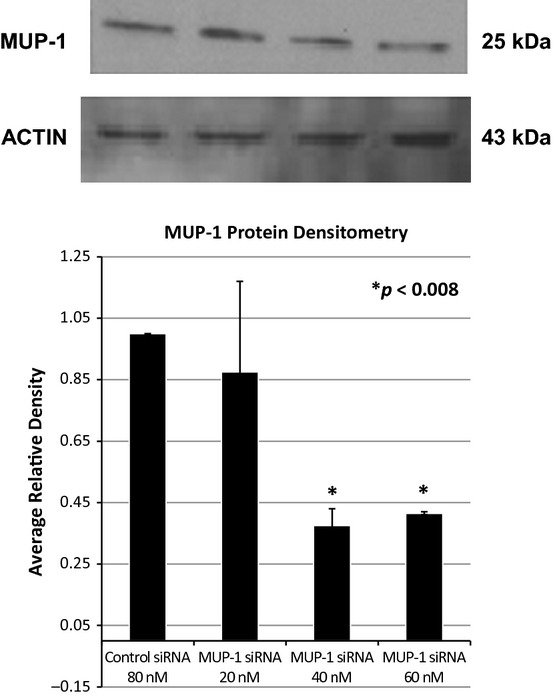
Western blot demonstrating silencing of MUP-1. Following their sixth passage, Cdx2-IEC cells were transfected with either MUP-1 siRNA (Thermo Scientific, Inc.) or Acell Control Non-Targeting siRNA (Thermo Scientific, Inc.). Western blot analysis was performed using MUP (F-3) mouse monoclonal antibody (Santa Cruz Biotechnology, Inc.).

**Figure 6:**
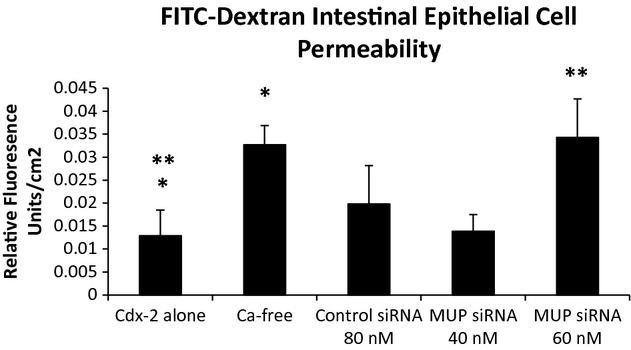
FITC-dextran permeability assay in Cdx2-IEC cells. Cells exposed to calcium-free (Ca-free) media resulted in a marked increase in cell permeability compared to Cdx2-IEC cells in control media (*P < 0.04). Cells treated with Control siRNA at 80 nmol/L and MUP-1 siRNA at 40 nmol/L concentrations were similar to untreated cells; whereas, cells treated with MUP-1 siRNA at 60 nmol/L concentration had significantly increased cell permeability compared to Cdx2-IEC cells in control media (**P < 0.0002). Indeed, permeability of cells treated with MUP-1 siRNA at 60 nmol/L concentration was similar to cells in calcium-free media.

Growth hormone treatment has beneficial effects on the intestine by normalizing intestinal permeability (Liu et al. 2003). We hypothesize that increased expression of MUPs by growth hormone activation (Kuhn et al. 1984) could contribute to this beneficial effect. We inhibited MUP-1 protein expression using siRNA technology. Following confirmation of inhibition of MUP-1 protein expression (Fig. 5), we performed a FITC-dextran permeability assay on Cdx2-IEC cells alone, Cdx2-IEC cells exposed to calcium-free media, Cdx2-IEC cells exposed to control siRNA, and Cdx2-IEC cells exposed to two different concentrations of MUP-1 siRNA (Fig. 6). As expected, cells exposed to calcium-free media resulted in a marked increase in cell permeability compared to Cdx2-IEC cells in control media (*P < 0.04). Cells treated with Control siRNA at 80 nmol/L and MUP-1 siRNA at 40 nmol/L concentrations were similar to untreated cells; whereas, cells treated with MUP-1 siRNA at 60 nmol/L concentration had significantly increased cell permeability compared to Cdx2-IEC cells in control media (**P < 0.0002). Indeed, permeability of cells treated with MUP-1 siRNA at 60 nmol/L concentration was similar to cells in calcium-free media.

## Discussion

Cholestasis can arise from a multitude of conditions in both the adult and pediatric population. In broad terms, cholestasis may result from biliary tract obstruction or hepatic parenchymal disease. Causes of biliary tract obstruction in the adult include pancreatic carcinoma, duodenal carcinoma and cholangiocarcinoma, benign biliary strictures, and choledochal cyst; whereas, biliary atresia and choledochal cyst are seen in infants and children. Causes of hepatic parenchymal disease in both age groups include hepatitis, total parenteral nutrition, sepsis, and other more rare cholestatic syndromes. In the neonatal population, cholestasis is commonly associated with a variety of intestinal pathologies, including necrotizing enterocolitis, intestinal atresias, midgut volvulus, gastroschisis, and Hirschsprung disease. In these conditions, a baby may not be able to tolerate much or any nutrition enterally. Total parenteral (intravenous) nutrition is required. As mentioned earlier, this type of nutrition causes cholestasis and the lack of enteral nutrition worsens the cholestasis; whereas, initiation of enteral nutrition and discontinuation of parenteral nutrition result in improvement of cholestatic liver dysfunction (Javid et al. 2005).

The goal of this study was to use microarray technology to generate candidate genes from both existing and novel pathways which play a role in the development of intestinal injury following cholestasis as modeled by CBDL. Using two different inbred strains of mice, A/J and C57Bl/6J, the design of this study had the benefit of not only discovering strain-independent but also strain-dependent changes in intestinal gene expression following cholestasis. More than 500 genes were increased by more than 2.0-fold following CBDL, and the following were identified as promising candidate genes for further study: MUPs, Serpina1a, and LCN-2.

Major urinary proteins (MUPs) comprise the lipocalin superfamily of lipophilic ligand carriers which, until recently, were thought to participate exclusively in pheromone function. In a seminal article from 2009, MUP1 was shown to be a regulator for glucose and lipid metabolism, in addition to its action as a pheromone ligand to mediate chemical signaling in mice (Zhou et al. 2009). MUP production is known to be increased by testosterone, thyroid hormone, and growth hormone (GH) (Kuhn et al. 1984). The latter hormone is well known to play a significant role in intestinal healing following injury. Increased growth of the small bowel mucosa has been demonstrated in mice overexpressing bovine growth hormone (Ulshen et al. 1993). Moreover, recombinant growth hormone significantly attenuated intestinal mucosal injuries and bacterial translocation in septic rats (Yi et al. 2007), possibly through a mechanism involving the rhGH inhibition of apoptosis of intestinal mucosa cells. Endotoxemia was also reduced after GH administration in jaundiced rats (Scopa et al. 2000). Some insight into the mechanism(s) by which GH exerts it effects have been elucidated in animal models. Prophylactic treatment with growth hormone promoted IgA secretion by B lymphocytes in the plasma and in the intestine in stressed postoperative rats (Ding et al. 2004). Similar effects have been seen in patients. GH was found to attenuate the depression in cellular immunity following surgical stress in a randomized, double-blind, controlled trial of 20 patients undergoing abdominal surgery (Liu et al. 2003). In addition, GH contributes to intestinal adaptation and has been documented to enable both adult and pediatric short gut syndrome patients to graduate from total parenteral nutrition (TPN) supplementation (Byrne et al. 1995; Velasco et al. 1999; Weiming et al. 2004). Growth hormone was also shown to reduce the increase in intestinal permeability seen following abdominal surgery (Liu et al. 2003). Consequently, when our microarray analysis demonstrated MUPs 1, 2, 3, 4, 7, 11, and 20 to be significantly increased following CBDL, we sought to explore this possible mechanism further. qPCR validated the microarray results and demonstrated a >200-fold increase in MUP expression in B6 sham mice compared to A/J sham mice. Most interestingly, the two strains of mice had different responses to CBDL. MUP expression increased >5-fold in A/J mice following CBDL; however, MUP expression in B6 mice decreased greatly following CBDL, such that there was no significant difference between two strains following CBDL**.** We speculate that GH rises in A/J mice following CBDL to increase MUP expression which, in turn, leads to increased intestinal resistance, strengthens the intestinal barrier, and helps limit injury; whereas, in B6 mice, GH reserves and MUP expression have been depleted and simply cannot keep up with the injury. Further work is needed to clarify this.

We documented baseline expression of MUP-1 in intestinal epithelial cells. Following transfection of these cells with MUP-1 siRNA at 60 nmol/L concentration, we noticed a marked increase in cell permeability. This finding implicates increased MUP expression leading to increased intestinal resistance as a mechanism potentially responsible for the beneficial effects of growth hormone therapy.

Serpina1a is a gene on Chromosome 14 that encodes alpha-1 antitrypsin, which is a type of serine protease inhibitor, serpin. Our microarray analysis demonstrated a markedly elevated level of Serpina1a gene expression in the B6 sham mice, as well as dramatic increases in Serpina1a gene expression following CBDL in both strains. qPCR confirmed these findings. B6 sham mice had approximately 50-fold increased levels of expression compared to A/J sham mice. Furthermore, both strains exhibited approximately 100-fold increased levels of expression following CBDL when compared to the A/J sham mice.

A novel mechanism was suggested by the work of Bacher et al. (1992). They found that in gastrointestinal cell lines, the formation of tight junctions involves cellular proteases which are susceptible to protease inhibitors. Moreover, the recent work by Buzza and colleagues (2010) showing that Matriptase, a membrane-type serine protease-1, regulated epithelial barrier formation and permeability in the intestine intrigued us. Using matriptase (*St14*) hypomorphic mice which express less than 1% of the gene product, Buzza and colleagues (2010) demonstrated these mice to have a leaky intestinal barrier with decreased TEER and increased paracellular permeability. This raised the hypothesis that matriptase (*St14*) hypomorphic mice would have a higher mortality following CBDL than wild-type B6 mice**.** The increased expression of Serpina1a, coupled with the decreased intestinal resistance that we had found following CBDL, suggested to us that matriptase (*St14*) hypomorphic mice with decreased intestinal resistance and leaky tight junctions would not tolerate CBDL and the resultant further inhibition of serine protease-1 as well as the wild-type B6 mice. To our surprise, we did not find a striking effect on mortality. This might be explained in part by the work of Beliveau et al. (2009) which showed that under in vitro conditions, Serpina1a was an impotent inhibitor of matriptase compared to other serpins, such as antithrombin III. Antithrombin III may very well inhibit matriptase in vivo; however, further experiments would be necessary to confirm this. Whether antithrombin III increases mortality in matriptase hypomorphic mice following CBDL might be confounded by the known salutary effects of antithrombin III in sepsis, including the attenuation of both hepatocyte apoptosis (Huang et al. 2010) and endotoxemia-induced healing impairment in the colon (Diller et al. 2009), as well as preserved mucosal thickness and villus height following CBDL (Caglikulekci et al. 2004). Nevertheless, our results suggest that matriptase does not play a significant role in the mortality following CBDL.

A potential mechanism behind the role of Serpina1a in the cholestatic intestine might be akin to that seen in studies of lung inflammation. Alpha-1 antitrypsin inhibits the enzyme, neutrophil elastase, which is released from neutrophils and macrophages during inflammation; neutrophil elastase destroys both bacteria and host tissue. Neutrophil elastase has also been shown to play a significant role in the pathogenesis of lung injury in pulmonary fibrosis (Yamanouchi et al. 1998). Moreover, alpha-1 antitrypsin was shown to induce hepatocyte growth factor (HGF) production by human lung fibroblasts and function as an anti-inflammatory and regenerative factor in addition to its role in protease inhibition (Kikuchi et al. 2000). HGF has previously been shown to increase intestinal epithelial cell mass and function in vivo (Kato et al. 1997) and also stimulate DNA content and protein content beyond the normal adaptive response following massive small intestinal resection (Kato et al. 1998). Our data suggest that increased expression of Serpina1a would increase HGF and provide a therapeutic approach to limit the intestinal injury following cholestasis, analogous to a recent study, in which alpha-1 antitrypsin therapy was shown to decrease intestinal permeability and ameliorate acute colitis and chronic ileitis in a murine model (Collins et al. 2013).

Lipocalin-2 is an iron-sequestering protein in the antibacterial innate immune response. Upon encountering invading bacteria, the Toll-like receptor 4 (TLR4) on immune cells stimulates the transcription, translation, and secretion of lipocalin-2 (Flo et al. 2004). It binds to siderophores secreted by pathogenic bacteria, including enterochelin secreted by *E. coli*, and prevents bacterial iron uptake (Flo et al. 2004). LCN2^−/−^ mice have decreased survival following *E. coli* infection compared to wild-type mice (Berger et al. 2006). Neutrophils isolated from LCN2^−/−^ mice showed significantly less bacteriostatic activity compared with wild-type controls. The bacteriostatic property of the wild-type neutrophils was abolished by the addition of exogenous iron, indicating that the main function of lipocalin-2 is to limit this essential element (Berger et al. 2006). Similarly, lipocalin-2 resistance confers an advantage to *Salmonella enterica* serotype Typhimurium for growth and survival in the inflamed intestine (Rafatellu et al. 2009). Our microarray analysis demonstrated significant increases in LCN2 following CBDL. qPCR confirmed this finding and is consistent with a strain-independent response to CBDL. LCN2 expression increased more than 20-fold and 40-fold, respectively, in A/J and B6 mice following CBDL. Increased LCN2 gene expression in the intestine following CBDL appears to be a mechanism whereby the host limits injury and mortality. This appears analogous to the increase in LCN2 gene expression coincident with an increase in epithelial cell apoptosis associated with intestinal adaptation following massive small bowel resection (Wildhaber et al. 2003). This is in agreement with earlier work by Devireddy et al. (2001) demonstrating lipocalin-2 to induce apoptosis in hematopoietic cells by an autocrine pathway. Thus, lipocalin-2 is a regulatory factor of intestinal growth.

In summary, our study has generated a resource of candidate genes for intestinal injury secondary to cholestasis. For Serpina1a, MUPs, and LCN2, some of the genes whose expression was most dramatically affected by CBDL, we confirmed the microarray results with qPCR. Novel mechanisms implicated by our results involve the growth hormone pathway, the acute phase response, and the innate immune response. More research is needed to further define these mechanisms; however, future therapeutic strategies might include: overexpression of Serpina1a and increased levels of HGF, upregulation of the GH pathway and increased MUP expression, and increased LCN2 expression to limit intestinal injury following cholestasis.

## Appendix

**Table A1:** List of all differentially expressed genes

Probe set ID	Gene accession	Gene symbol	Gene description	Cytoband	AJ CBDL vs sham FC	AJ CBDL vs sham FDR	B6 CBDL vs sham FC	B6 CBDL vs sham FDR
10566477	NM_017371	**Hpx**	hemopexin	7 F1	**37.4012**	**1.00E−04**	**4.7408**	**0.0435**
10581605	NM_017370	**Hp**	haptoglobin	8 D3|8 55.0 cM	**34.152**	**3.00E−04**	**6.662**	**0.0445**
10505438	NM_008768	**Orm1**	orosomucoid 1	4 B3|4 31.4 cM	**18.2062**	**2.00E−04**	**4.3826**	**0.0404**
10414192	NM_133653	**Mat1a**	methionine adenosyltransferase I, alpha	14 C1	**17.981**	**0.001**	**7.268**	**0.0306**
10511886	NM_016668 // NM_016668	**Bhmt // Bhmt**	betaine-homocysteine methyltransferase // betaine-homocysteine methyltransferase	13 D1 // 13 D1	**17.9485**	**0.0014**	**10.4211**	**0.0197**
10457114	NM_016668	**Bhmt**	betaine-homocysteine methyltransferase	13 D1	**15.6549**	**5.00E−04**	**8.3998**	**0.0109**
10578352	NM_145594	**Fgl1**	fibrinogen-like protein 1	8 A4	**13.8891**	**1.00E−04**	**4.1148**	**0.0178**
10575349	NM_146214	**Tat**	tyrosine aminotransferase	8 D3	**12.4752**	**3.00E−04**	**4.323**	**0.0294**
10449452	NM_010220	**Fkbp5**	FK506 binding protein 5	17 A3.3|17 13.0 cM	**11.8521**	**0.0015**	**6.359**	**0.0294**
10490989	NM_001042611	**Cp**	ceruloplasmin	3 D	**11.1162**	**0**	**3.3852**	**0.0109**
10481627	NM_008491	**Lcn2**	lipocalin 2	2 A3|2 27.0 cM	**9.7186**	**8.00E−04**	**8.0914**	**0.0098**
10360328	NM_011318	**Apcs**	serum amyloid P-component	1 H3|1 94.2 cM	**9.2166**	**0**	**3.1253**	**0.0123**
10451953	NM_029796	**Lrg1**	leucine-rich alpha-2-glycoprotein 1	17 D|17 10.0 cM	**6.9919**	**6.00E−04**	**5.0051**	**0.0109**
10574023	NM_008630	**Mt2**	metallothionein 2	8 C5|8 45.0 cM	**6.9697**	**0.0206**	**8.4549**	**0.0307**
10515187	NM_007822	**Cyp4a14**	cytochrome P450, family 4, subfamily a, polypeptide 14	4 D1|4 49.5 cM	**4.6609**	**0.0378**	**9.8282**	**0.015**
10418434	NM_008407	**Itih3**	inter-alpha trypsin inhibitor, heavy chain 3	14 A2-C1	**4.2071**	**9.00E−04**	**4.0594**	**0.0078**
10543017	NM_013743	**Pdk4**	pyruvate dehydrogenase kinase, isoenzyme 4	6 A1|6 0.63 cM	**3.5193**	**0.0145**	**3.492**	**0.0368**
10503520	NM_015767	**Ttpa**	tocopherol (alpha) transfer protein	4 A3|4 22.7 cM	**3.3338**	**0.003**	**2.5756**	**0.0354**
10492748	NM_010196	**Fga**	fibrinogen alpha chain	3 F1|3 44.8 cM	**3.1077**	**0.0013**	**2.2512**	**0.0307**
10496727	NM_026993	**Ddah1**	dimethylarginine dimethylaminohydrolase 1	3 H3	**3.0694**	**0.001**	**2.967**	**0.0098**
10583732	NM_010700	**Ldlr**	low density lipoprotein receptor	9 A3|9 5.0 cM	**3.0527**	**0**	**2.0755**	**0.0024**
10507177	NM_001100181	**Cyp4a32**	cytochrome P450, family 4, subfamily a, polypeptide 32	4 D1	**2.7853**	**0.0347**	**3.3932**	**0.0344**
10527920	NM_020010	**Cyp51**	cytochrome P450, family 51	5 A2|5 1.2 cM	**2.7708**	**0.001**	**2.3088**	**0.015**
10362073	NM_001161845	**Sgk1**	serum/glucocorticoid regulated kinase 1	10 A3	**2.7412**	**0.0073**	**3.4689**	**0.0109**
10379153	NM_009657	**Aldoc**	aldolase C, fructose-bisphosphate	11 B5|11 44.98 cM	**2.699**	**0.0096**	**2.6246**	**0.0301**
10420730	NM_010191	**Fdft1**	farnesyl diphosphate farnesyl transferase 1	14 D1|14 3.0 cM	**2.6151**	**0.001**	**2.0228**	**0.0228**
10515201	NM_007823	**Cyp4b1**	cytochrome P450, family 4, subfamily b, polypeptide 1	4 D1|4 49.5 cM	**2.5614**	**0.001**	**1.7862**	**0.0445**
10568001	NM_133670	**Sult1a1**	sulfotransferase family 1A, phenol-preferring, member 1	7 F3|7 4.0 cM	**2.5199**	**1.00E−04**	**1.7899**	**0.0109**
10520362	NM_153526	**Insig1**	insulin induced gene 1	5 B1	**2.4631**	**0.0014**	**1.836**	**0.0451**
10412909	NM_010191	**Fdft1**	farnesyl diphosphate farnesyl transferase 1	14 D1|14 3.0 cM	**2.4306**	**6.00E−04**	**2.072**	**0.0109**
10424349	NM_009270	**Sqle**	squalene epoxidase	15 D1	**2.3683**	**0.0023**	**1.9609**	**0.0307**
10478048	NM_008489	**Lbp**	lipopolysaccharide binding protein	2 H1|2 83.0 cM	**2.3404**	**6.00E−04**	**1.8161**	**0.0194**
10499483	NM_134469	**Fdps**	farnesyl diphosphate synthetase	3 F1|3 42.6 cM	**2.2316**	**0.0044**	**2.2171**	**0.017**
10412466	NM_145942	**Hmgcs1**	3-hydroxy-3-methylglutaryl -Coenzyme A synthase 1	—	**2.213**	**0.0012**	**1.7749**	**0.0294**
10364194	NM_146006	**Lss**	lanosterol synthase	10 C1|10 41.1 cM	**2.1256**	**0.0051**	**1.9021**	**0.0346**
10378793	NM_025655	**Tmigd1**	transmembrane and immunoglobulin domain containing 1	11 B5	**2.0643**	**0.0316**	**3.0221**	**0.0109**
10365830	—	**—**	—	—	**2.0533**	**0.0032**	**1.9576**	**0.0178**
10542470	NM_019946	**Mgst1**	microsomal glutathione S-transferase 1	6 G1	**1.9951**	**0.0124**	**1.9203**	**0.0416**
10506571	NM_053272	**Dhcr24**	24-dehydrocholesterol reductase	4 C7	**1.8836**	**0.0014**	**1.8709**	**0.0109**
10450242	NM_009780	**C4b**	complement component 4B (Childo blood group)	17 B1|17 18.8 cM	**1.8607**	**0.0124**	**1.849**	**0.0333**
10585942	NM_001033498	**Gramd2**	GRAM domain containing 2	9 B	**1.8264**	**0.0037**	**1.622**	**0.0375**
10493548	NM_026784	**Pmvk**	phosphomevalonate kinase	3 F1	**1.7852**	**0.0105**	**1.808**	**0.0273**
10492964	NM_009690	**Cd5l**	CD5 antigen-like	3 F1	**1.6284**	**0.0195**	**1.8539**	**0.0169**
10524555	NM_023556	**Mvk**	mevalonate kinase	5 F|5 64.0 cM	**1.6043**	**0.0144**	**1.63**	**0.0307**
10600082	NM_010941	**Nsdhl**	NAD(P) dependent steroid dehydrogenase-like	X A7.3|X 28.87 cM	**1.5727**	**0.009**	**1.7157**	**0.0116**
10573626	NM_173866	**Gpt2**	glutamic pyruvate transaminase (alanine aminotransferase) 2	8 C3	**1.5293**	**0.0438**	**1.6633**	**0.0425**
10526630	NM_015799	**Trfr2**	transferrin receptor 2	5 G2	**1.5175**	**0.0197**	**1.5238**	**0.0435**
10456699	NM_177470	**Acaa2**	acetyl-Coenzyme A acyltransferase 2 (mitochondrial 3-oxoacy l-Coenzyme A thiolase)	18 E2|18 45.0 cM	**1.5095**	**0.029**	**1.6537**	**0.0273**
10393970	NM_007988	**Fasn**	fatty acid synthase	11 E2|11 72.0 cM	**1.4769**	**0.004**	**1.4846**	**0.015**
10525893	NM_030210	**Aacs**	acetoacetyl-CoA synthetase	5 F	**1.4673**	**0.0242**	**1.4896**	**0.0456**
10425695	NM_033218	**Srebf2**	sterol regulatory element binding factor 2	15 E1	**1.4633**	**0.0095**	**1.4826**	**0.0233**
10569972	NM_026058	**Lass4**	LAG1 homolog, ceramide synthase 4	8 A1.2	**1.4318**	**0.0145**	**1.5**	**0.0228**
10570437	NM_025785	**Fbxo25**	F-box protein 25	8 A1.1	**−1.3809**	**0.0356**	**−1.4953**	**0.0294**
10447239	NM_026180	**Abcg8**	ATP-binding cassette, sub-family G (WHITE), member 8	17 E4|17 54.5 cM	**−1.5584**	**0.013**	**−1.8003**	**0.0109**
10425945	NM_010180	**Fbln1**	fibulin 1	15 E-F	**−1.5672**	**0.013**	**−1.601**	**0.0287**
10453318	NM_031884	**Abcg5**	ATP-binding cassette, sub-family G (WHITE), member 5	17 E4|17 54.5 cM	**−1.6829**	**0.0069**	**−1.6878**	**0.0224**
10390032	NM_153807	**Acsf2**	acyl-CoA synthetase family member 2	11 D	**−1.8821**	**0.0041**	**−2.228**	**0.0078**
10492426	NM_153807	**Acsf2**	acyl-CoA synthetase family member 2	11 D	**−1.9567**	**0.0045**	**−2.1236**	**0.0109**
10518674	NM_022020	**Rbp7**	retinol binding protein 7, cellular	4 E2	**−2.0739**	**0.0021**	**−1.8152**	**0.0258**
10462442	NM_001164724	**Il33**	interleukin 33	19 C2	**−2.0935**	**0.0078**	**−1.9454**	**0.0354**
10575833	NM_008290	**Hsd17b2**	hydroxysteroid (17-beta) dehydrogenase 2	8 E1	**−2.2055**	**0.0025**	**−1.9459**	**0.026**
10577655	NM_008324	**Ido1**	indoleamine 2,3-dioxygenase 1	8 A2	**−2.4459**	**0.0461**	**−3.4593**	**0.0249**
10463005	NM_028089	**Cyp2c55**	cytochrome P450, family 2, subfamily c, polypeptide 55	19 C3	**−2.5559**	**0.0458**	**−14.6416**	**4.00E−04**
10373334	NM_013786	**Hsd17b6**	hydroxysteroid (17-beta) dehydrogenase 6	10 D3	**−2.6186**	**2.00E−04**	**−1.7122**	**0.0241**
10502214	NM_027816	**Cyp2u1**	cytochrome P450, family 2, subfamily u, polypeptide 1	3 H1	**−3.4307**	**1.00E−04**	**-1.6652**	**0.0425**
10514912	NM_007860	**Dio1**	deiodinase, iodothyronine, type I	4 C7|4 48.7 cM	**−4.5064**	**0.0012**	**−3.8938**	**0.0109**
10480155	NM_001081084	**Cubn**	cubilin (intrinsic factor -cobalamin receptor)	2 A1|2 9.0 cM	**−4.8123**	**3.00E−04**	**−2.7793**	**0.016**
10585749	NM_009992	**Cyp1a1**	cytochrome P450, family 1, subfamily a, polypeptide 1	9 B|9 31.0 cM	**−5.6507**	**0.001**	**−3.3135**	**0.0301**
10513420	NM_001134675	**Mup7**	major urinary protein 7	4 B3|4	**127.8618**	**0.001**		
10513455	NM_008647	**Mup2**	major urinary protein 2	4 B3	**126.9789**	**0.001**		
10513437	NM_001164526	**Mup11**	major urinary protein 11	4 B3	**117.1801**	**0.001**		
10513428	NM_001045550	**Mup2**	major urinary protein 2	4 B3	**113.5703**	**0.001**		
10513472	NM_008647	**Mup2**	major urinary protein 2	4 B3	**104.5563**	**0.001**		
10513504	NM_001045550	**Mup2**	major urinary protein 2	4 B3	**100.7876**	**0.001**		
10513497	NM_001045550	**Mup2**	major urinary protein 2	4 B3	**94.2911**	**0.001**		
10513521	NM_001012323	**Mup20**	major urinary protein 20	4 B3	**93.1978**	**0.0021**		
10513467	NM_001045550	**Mup2**	major urinary protein 2	4 B3	**90.6275**	**0.001**		
10513512	NM_001163011	**Mup1**	major urinary protein 1	4 B3|4 27.8 cM	**73.9991**	**0.001**		
10523062	NM_009654	**Alb**	albumin	5 E1|5 50.0 cM	**61.2531**	**4.00E−04**		
10434689	NM_013465	**Ahsg**	alpha-2-HS-glycoprotein	16 B1|16 15.0 cM	**58.1197**	**3.00E−04**		
10531149	NM_008096	**Gc**	group specific component	5 E1|5 44.0 cM	**52.3423**	**1.00E−04**		
10492735	NM_133862	**Fgg**	fibrinogen gamma chain	3 E3|3 41.3 cM	**38.7526**	**1.00E−04**		
10498981	NM_181849	**Fgb**	fibrinogen beta chain	3 E3|3 48.2 cM	**38.2336**	**1.00E−04**		
10402406	NM_009245	**Serpina1c**	serine (or cysteine) peptidase inhibitor, clade A, member 1C	12 E|12 51.0 cM	**30.3389**	**3.00E−04**		
10402399	NM_009243	**Serpina1a**	serine (or cysteine) peptidase inhibitor, clade A, member 1A	12 E|12 51.0 cM	**23.9693**	**3.00E−04**		
10558673	NM_021282	**Cyp2e1**	cytochrome P450, family 2, subfamily e, polypeptide 1	7 F5|7 68.4 cM	**21.9342**	**0.0012**		
10562169	NM_032541	**Hamp**	hepcidin antimicrobial peptide	7 B1|7 11.0 cM	**21.1349**	**0.004**		
10434719	NM_001102411	**Kng1**	kininogen 1	16 B1	**19.6656**	**7.00E−04**		
10402409	NM_009247	**Serpina1e**	serine (or cysteine) peptidase inhibitor, clade A, member 1E	12 E	**18.9172**	**3.00E−04**		
10548207	NM_007376	**Pzp**	pregnancy zone protein	6 F1-G3|6 62.0 cM	**18.3515**	**3.00E−04**		
10402390	NM_009244	**Serpina1b**	serine (or cysteine) preptidase inhibitor, clade A, member 1B	12 E|12 51.0 cM	**15.5285**	**5.00E−04**		
10498921	NM_019911	**Tdo2**	tryptophan 2,3-dioxygenase	3 E3	**13.9226**	**0.0023**		
10596148	NM_133977	**Trf**	transferrin	9 F1-F3|9 56.0 cM	**13.3568**	**4.00E−04**		
10382189	NM_013475	**Apoh**	apolipoprotein H	11 D|11 63.0 cM	**13.1193**	**0.001**		
10553274	NM_011314	**Saa2**	serum amyloid A 2	7 B4|7 23.5 cM	**10.9174**	**0.015**		
10541480	NR_027619	**Mug-ps1**	murinoglobulin, pseudogene 1	6 F1	**10.2599**	**0.0013**		
10496825	NM_009474	**Uox**	urate oxidase	3 H2|3 75.0 cM	**10.1685**	**0.0046**		
10497337	NM_009799	**Car1**	carbonic anhydrase 1	3 A1|3 10.5 cM	**9.9909**	**0.0175**		
10454192	NM_013697	**Ttr**	transthyretin	18 A2|18 7.0 cM	**9.9844**	**0.0025**		
10463037	NM_010003	**Cyp2c39**	cytochrome P450, family 2, subfamily c, polypeptide 39	19 C3	**9.7038**	**1.00E−04**		
10398060	NM_011458	**Serpina3k**	serine (or cysteine) peptidase inhibitor, clade A, member 3K	12 E|12 51.5 cM	**9.5497**	**0.0042**		
10541448	NR_027619	**Mug-ps1**	murinoglobulin, pseudogene 1	6 F1	**9.4334**	**0.0027**		
10541410	NM_008645	**Mug1**	murinoglobulin 1	6 F1	**9.3764**	**0.001**		
10379190	NM_011707	**Vtn**	vitronectin	11 B5|11 45.09 cM	**8.9998**	**0.0014**		
10431915	NM_027052	**Slc38a4**	solute carrier family 38, member 4	15 F1	**8.9956**	**0.0027**		
10351546	NM_013474	**Apoa2**	apolipoprotein A-II	1 H3|1 92.6 cM	**8.8013**	**8.00E−04**		
10467410	NM_145499	**Cyp2c70**	cytochrome P450, family 2, subfamily c, polypeptide 70	19 C3	**8.4844**	**0.0014**		
10467319	NM_001159487	**Rbp4**	retinol binding protein 4, plasma	19 D1|19 38.0 cM	**7.858**	**0.0021**		
10542983	NM_011134	**Pon1**	paraoxonase 1	6 A2|6 0.5 cM	**7.6708**	**0.021**		
10560618	NM_007469	**Apoc1**	apolipoprotein C-I	7 A3|7 4.0 cM	**7.6693**	**0.0065**		
10398075	NM_009252	**Serpina3n**	serine (or cysteine) peptidase inhibitor, clade A, member 3N	12 F1	**7.4314**	**0.0115**		
10368343	NM_007482	**Arg1**	arginase, liver	10 A4	**7.3671**	**9.00E−04**		
10563611	NM_009117	**Saa1**	serum amyloid A 1	7 B4|7 23.5 cM	**7.1769**	**0.0095**		
10434709	NM_053176	**Hrg**	histidine-rich glycoprotein	16 B1|16 14.1 cM	**7.0319**	**0.0028**		
10513529	NM_001039544	**Mup3**	major urinary protein 3	4 B3	**6.9857**	**0.0286**		
10549154	BC151094	**Gm766**	predicted gene 766	6 G3	**6.9004**	**0.0267**		
10413615	NM_018746	**Itih4**	inter alpha-trypsin inhibitor, heavy chain 4	14 B|14 11.75 cM	**6.7951**	**0.0012**		
10441753	NM_008877	**Plg**	plasminogen	17 A1|17 7.3 cM	**6.266**	**0.0022**		
10401289	NM_001177561	**Slc10a1**	solute carrier family 10 (sodium/bile acid cotransporter family), member 1	12 D1|12 37.0 cM	**6.0785**	**0.0028**		
10485027	NM_010168	**F2**	coagulation factor II	2 E1|2 47.5 cM	**6.048**	**0.0096**		
10402394	NM_009246	**Serpina1d**	serine (or cysteine) peptidase inhibitor, clade A, member 1D	12 E|12 51.0 cM	**6.0378**	**0.0135**		
10363860	NM_025807	**Slc16a9**	solute carrier family 16 (monocarboxylic acid transporters), member 9	10 B5.3	**5.8753**	**0.0065**		
10434698	NM_021564	**Fetub**	fetuin beta	16 B|16 14.1 cM	**5.7467**	**0.0098**		
10580635	NM_053200	**Ces3**	carboxylesterase 3	8 C5	**5.6566**	**0.0021**		
10526712	NM_013478	**Azgp1**	alpha-2-glycoprotein 1, zinc	5 G2|5 78.0 cM	**5.4824**	**0.0232**		
10506125	NM_013913	**Angptl3**	angiopoietin-like 3	4 C6|4 48.0 cM	**5.2732**	**0.0124**		
10580624	NM_007954	**Es1**	esterase 1	8 C5|8 43.0 cM	**5.1268**	**0.0355**		
10513630	NM_007443	**Ambp**	alpha 1 microglobulin/bikunin	4 C1-C3|4 30.6 cM	**5.1009**	**0.0025**		
10351852	NM_007768	**Crp**	C-reactive protein, pentraxin-related	1 H3|1 94.2 cM	**4.9882**	**0.0025**		
10363541	NM_007494	**Ass1**	argininosuccinate synthetase 1	2 B|2 20.0 cM	**4.9534**	**0.0057**		
10480003	NM_010582	**Itih2**	inter-alpha trypsin inhibitor, heavy chain 2	2 A1|2 1.0 cM	**4.9513**	**0.0059**		
10542615	NM_020495	**Slco1b2**	solute carrier organic anion transporter family, member 1b2	6 G1|6 67.0 cM	**4.89**	**0.0212**		
10367221	NM_133997	**Apof**	apolipoprotein F	10 D3|10 73.0 cM	**4.8785**	**0.0025**		
10551287	NM_133657	**Cyp2a12**	cytochrome P450, family 2, subfamily a, polypeptide 12	7 A3	**4.8046**	**0.0096**		
10494643	NM_008256	**Hmgcs2**	3-hydroxy-3-methylglutaryl -Coenzyme A synthase 2	3 F2.2|3 48.0 cM	**4.5815**	**0.0144**		
10563602	NM_011316	**Saa4**	serum amyloid A 4	7 B4|7 23.5 cM	**4.5245**	**0.001**		
10471154	NM_007494	**Ass1**	argininosuccinate synthetase 1	2 B|2 20.0 cM	**4.4979**	**0.0069**		
10357516	NM_007576	**C4bp**	complement component 4 binding protein	1 E4|1 67.6 cM	**4.4217**	**0.001**		
10416451	NM_019775	**Cpb2**	carboxypeptidase B2 (plasma)	14 D2	**4.3546**	**0.0036**		
10551293	NM_007817	**Cyp2f2**	cytochrome P450, family 2, subfamily f, polypeptide 2	7 A3	**4.2794**	**0.0394**		
10398069	NM_009253	**Serpina3m**	serine (or cysteine) peptidase inhibitor, clade A, member 3M	12 E	**4.2775**	**0.0231**		
10438681	NM_201375	**Kng2**	kininogen 2	16 B1	**4.2464**	**0.0051**		
10367215	NM_133996	**Apon**	apolipoprotein N	10 D3|10 76.0 cM	**4.2277**	**0.0021**		
10351015	NM_080844	**Serpinc1**	serine (or cysteine) peptidase inhibitor, clade C (antithrombin), member 1	1 H2.1|1 84.6 cM	**4.1985**	**0.0034**		
10403322	NM_030611	**Akr1c6**	aldo-keto reductase family 1, member C6	13 A2|13 8.0 cM	**4.1493**	**0.0498**		
10452316	NM_009778	**C3**	complement component 3	17 E1-E3|17 34.3 cM	**4.1151**	**0.0339**		
10596718	NM_023805	**Slc38a3**	solute carrier family 38, member 3	9 F1|9 63.0 cM	**4.1024**	**0.0239**		
10501555	NM_007446	**Amy1**	amylase 1, salivary	3 F3|3 50.0 cM	**4.1021**	**0.0069**		
10519497	NM_054098	**Steap4**	STEAP family member 4	5 A1	**3.9917**	**0.0063**		
10496001	NM_007686	**Cfi**	complement component factor i	3 G3|3 66.6 cM	**3.9698**	**0.0014**		
10475532	NM_021507	**Sqrdl**	sulfide quinone reductase-like (yeast)	2 F2	**3.9595**	**0**		
10593225	NM_001033324	**Zbtb16**	zinc finger and BTB domain containing 16	9 A5.3|9 23.0 cM	**3.8909**	**0.018**		
10458828	NM_033037	**Cdo1**	cysteine dioxygenase 1, cytosolic	18 C|18 23.0 cM	**3.7452**	**0.0025**		
10514763	NM_146148	**C8a**	complement component 8, alpha polypeptide	4 C6	**3.7375**	**0.0096**		
10358339	NM_009888	**Cfh**	complement component factor h	1 F|1 74.1 cM	**3.6868**	**0.0032**		
10514532	NM_010007	**Cyp2j5**	cytochrome P450, family 2, subfamily j, polypeptide 5	4 C5|4 46.5 cM	**3.6645**	**0.0256**		
10450038	NM_020581	**Angptl4**	angiopoietin-like 4	17 B1	**3.5851**	**0.0062**		
10511375	NM_007824	**Cyp7a1**	cytochrome P450, family 7, subfamily a, polypeptide 1	4 A1	**3.5797**	**0.0076**		
10423002	NM_207216	**Ugt3a1**	UDP glycosyltransferases 3 family, polypeptide A1	15 A1	**3.5454**	**0.0345**		
10483410	NM_021022	**Abcb11**	ATP-binding cassette, sub-family B (MDR/TAP), member 11	2 C2|2 38.4 cM	**3.4306**	**0.0445**		
10447885	NM_153151	**Acat3**	acetyl-Coenzyme A acetyltransferase 3	17 A1|17 7.55 cM	**3.3695**	**0.0023**		
10537169	NM_009731	**Akr1b7**	aldo-keto reductase family 1, member B7	6 B1|6 14.0 cM	**3.3685**	**0.0032**		
10395273	BC052902	**Gdap10**	ganglioside-induced differentiation-associated-protein 10	12 A3	**3.3561**	**0.0043**		
10503502	NM_015767	**Ttpa**	tocopherol (alpha) transfer protein	4 A3|4 22.7 cM	**3.2169**	**0.0089**		
10585015	NM_080434	**Apoa5**	apolipoprotein A-V	9 B	**3.2153**	**0.0026**		
10581388	NM_008490	**Lcat**	lecithin cholesterol acyltransferase	8 D3|8 53.0 cM	**3.2103**	**0.0014**		
10482004	BC094504	**AI182371**	expressed sequence AI182371	2 B	**3.1837**	**0.0281**		
10574027	NM_013602	**Mt1**	metallothionein 1	8 C5|8 45.0 cM	**3.1654**	**0.0374**		
10379736	NM_183249	**1100001G20Rik**	RIKEN cDNA 1100001G20 gene	11 C	**3.1049**	**0.0151**		
10420899	NM_178747	**Gulo**	gulonolactone (L-) oxidase	14 D1	**3.0186**	**0.0345**		
10435626	NM_013547	**Hgd**	homogentisate 1, 2-dioxygenase	16 B3|16 27.3 cM	**2.9656**	**0.0296**		
10345065	NM_001077353	**Gsta3**	glutathione S-transferase, alpha 3	1 A4|1 15.0 cM	**2.9309**	**0.0145**		
10356145	NM_030556	**Slc19a3**	solute carrier family 19 (sodium/hydrogen exchanger), member 3	1 C5|1 51.0 cM	**2.9123**	**0.0301**		
10533612	NM_008277	**Hpd**	4-hydroxyphenylpyruvic acid dioxygenase	5 F|5 67.0 cM	**2.8602**	**0.0373**		
10451451	NM_010321	**Gnmt**	glycine N-methyltransferase	17 C|17 10.0 cM	**2.8319**	**0.0461**		
10543333	NM_013930	**Aass**	aminoadipate-semialdehyde synthase	6 A3.1|6 4.5 cM	**2.8069**	**0.0115**		
10596166	NM_027918	**1300017J02Rik**	RIKEN cDNA 1300017J02 gene	9 F1	**2.7756**	**0.0308**		
10406646	NM_028772	**Dmgdh**	dimethylglycine dehydrogenase precursor	13 C3	**2.7336**	**0.0244**		
10500547	NM_153193	**Hsd3b2**	hydroxy-delta-5-steroid dehydrogenase, 3 beta- and steroid delta-isomerase 2	3 F2.2|3 49.1 cM	**2.6399**	**0.0032**		
10570717	NM_001177522	**Gm14850**	predicted gene 14850	8 A2	**2.5973**	**0.0148**		
10361234	NM_008288	**Hsd11b1**	hydroxysteroid 11-beta dehydrogenase 1	1 H6	**2.5957**	**0.0096**		
10507171	NM_201640	**Cyp4a31**	cytochrome P450, family 4, subfamily a, polypeptide 31	4 D1	**2.575**	**0.0484**		
10570732	NM_001177528	**Gm15315**	predicted gene 15315	8 A2|8	**2.5592**	**0.0098**		
10599826	NM_007979	**F9**	coagulation factor IX	X A6-A7|X 22.0 cM	**2.5474**	**0.0143**		
10578916	NM_025436	**Sc4mol**	sterol-C4-methyl oxidase-like	8 B3.1	**2.5379**	**0.0023**		
10399365	NM_177802	**Slc7a15**	solute carrier family 7 (cationic amino acid transporter, y+ system), member 15	12 A1.1	**2.5357**	**0.0361**		
10570693	NM_007851	**Defa5**	defensin, alpha, 5	8 A2	**2.5181**	**0.0129**		
10497590	NM_007963	**Mecom**	MDS1 and EVI1 complex locus	3 A3|3 14.4 cM	**2.5047**	**0.0091**		
10545881	NM_053096	**Cml2**	camello-like 2	6 C3	**2.5013**	**0.027**		
10478523	—	**—**	—	—	**2.4962**	**0.0021**		
10454944	NM_201256	**Eif4ebp3**	eukaryotic translation initiation factor 4E binding protein 3	18 B2	**2.4951**	**0.0117**		
10386683	NM_026183	**Slc47a1**	solute carrier family 47, member 1	11 B2	**2.4534**	**0.0067**		
10365559	NM_010512	**Igf1**	insulin-like growth factor 1	10 C1|10 48.0 cM	**2.4375**	**0.0041**		
10500555	NM_001161742	**Hsd3b3**	hydroxy-delta-5-steroid dehydrogenase, 3 beta- and steroid delta-isomerase 3	3 F2.2|3 49.1 cM	**2.4177**	**0.0094**		
10513412	NM_008648	**Mup4**	major urinary protein 4	4 B3|4 27.8 cM	**2.3986**	**5.00E−04**		
10491846	—	**—**	—	—	**2.3936**	**0.0128**		
10537306	NM_145364	**Akr1d1**	aldo-keto reductase family 1, member D1	6 B1	**2.3924**	**0.0045**		
10589099	NM_029634	**Ip6k2**	inositol hexaphosphate kinase 2	9 F2	**2.3776**	**0.0021**		
10594825	NM_022026	**Aqp9**	aquaporin 9	9 D	**2.3723**	**0.0412**		
10519578	NM_008830	**Abcb4**	ATP-binding cassette, sub-family B (MDR/TAP), member 4	5 A1|5 1.0 cM	**2.3672**	**0.0297**		
10590957	NM_010242	**Fut4**	fucosyltransferase 4	9 A2|9 3.0 cM	**2.3611**	**0.0019**		
10447317	NM_010137	**Epas1**	endothelial PAS domain protein 1	17 E4	**2.2998**	**0.0142**		
10352000	NM_133809	**Kmo**	kynurenine 3-monooxygenase (kynurenine 3-hydroxylase)	1 H4	**2.2966**	**0.0301**		
10360840	NM_001081361	**Mosc1**	MOCO sulphurase C-terminal domain containing 1	—	**2.2607**	**0.0105**		
10570660	NM_001177481	**Gm10104**	predicted gene 10104	8 A2|8	**2.2377**	**0.0163**		
10386460	NM_008819	**Pemt**	phosphatidylethanolamine N-methyltransferase	11 B1.3|11 31.0 cM	**2.2274**	**0.0265**		
10576901	NM_011388	**Slc10a2**	solute carrier family 10, member 2	8 A1.1|8 2.0 cM	**2.2212**	**0.0253**		
10530319	NM_001038999	**Atp8a1**	ATPase, aminophospholipid transporter (APLT), class I, type 8A, member 1	5 C3.1	**2.1827**	**0.0152**		
10502050	NM_027808	**Alpk1**	alpha-kinase 1	3 H1	**2.1604**	**0.04**		
10381096	NM_010517	**Igfbp4**	insulin-like growth factor binding protein 4	11 D	**2.1452**	**0.0025**		
10408689	NM_153529	**Nrn1**	neuritin 1	13 A3.3	**2.1125**	**0.0301**		
10587266	NM_010295	**Gclc**	glutamate-cysteine ligase, catalytic subunit	9 D-E|9 42.0 cM	**2.1067**	**0.0473**		
10429588	NM_001039720	**9030619P08Rik**	RIKEN cDNA 9030619P08 gene	15 D3	**2.1009**	**0.0025**		
10537146	NM_008012	**Akr1b8**	aldo-keto reductase family 1, member B8	6 B1|6 13.0 cM	**2.0926**	**0.0397**		
10576774	NM_029465	**Clec4g**	C-type lectin domain family 4, member g	8 A1.1	**2.0888**	**0.0367**		
10542592	NR_033555	**Gm10400**	predicted gene 10400	—	**2.0729**	**0.0104**		
10496605	NM_173763	**Ccbl2**	cysteine conjugate-beta lyase 2	3 H1	**2.0685**	**0.0222**		
10358299	NM_001029977	**EG214403**	predicted gene, EG214403	1 F	**2.0607**	**0.0386**		
10494023	NM_011281	**Rorc**	RAR-related orphan receptor gamma	3 F2	**2.0501**	**0.0013**		
10357363	NM_001081756	**Nckap5**	NCK-associated protein 5	1 E3	**2.0191**	**0.023**		
10506452	AY512949	**AY512949**	cDNA sequence AY512949	—	**2.0161**	**0.0416**		
10512895	NM_007519	**Baat**	bile acid-Coenzyme A: amino acid N-acyltransferase	4 B1|4 22.7 cM	**2.0089**	**0.0204**		
10388430	NM_011340	**Serpinf1**	serine (or cysteine) peptidase inhibitor, clade F, member 1	11 B5	**2.0036**	**0.0181**		
10454198	NM_026301	**Rnf125**	ring finger protein 125	18 A2	**2.003**	**0.0053**		
10606989	NM_001077364	**Tsc22d3**	TSC22 domain family, member 3	X F1	**1.9909**	**0.0197**		
10418455	NM_008406	**Itih1**	inter-alpha trypsin inhibitor, heavy chain 1	14 A2-C1	**1.9848**	**0.0374**		
10365482	NM_011595	**Timp3**	tissue inhibitor of metalloproteinase 3	10 C1-D1|10 47.0 cM	**1.9692**	**0.0355**		
10388440	NM_008878	**Serpinf2**	serine (or cysteine) peptidase inhibitor, clade F, member 2	11 B5	**1.9646**	**0.043**		
10372682	NM_029875	**Slc35e3**	solute carrier family 35, member E3	10 D2	**1.9641**	**0.0062**		
10571657	NM_007981	**Acsl1**	acyl-CoA synthetase long-chain family member 1	8 B2	**1.9589**	**0.0041**		
10581650	NM_011998	**Chst4**	carbohydrate (chondroitin 6/keratan) sulfotransferase 4	—	**1.9469**	**0.039**		
10394990	NM_026037	**Mboat2**	membrane bound O-acyltransferase domain containing 2	12 A1.3	**1.9401**	**0.0244**		
10596072	NM_001161362	**Ppp2r3a**	protein phosphatase 2, regulatory subunit B'', alpha	9 E4	**1.9318**	**0.0021**		
10411332	NM_008255	**Hmgcr**	3-hydroxy-3-methylglutaryl-Coenzyme A reductase	13 D1|13 49.0 cM	**1.9292**	**0.0013**		
10571840	NM_008278	**Hpgd**	hydroxyprostaglandin dehydrogenase 15 (NAD)	8 B3.2	**1.9235**	**0.0025**		
10435787	—	**—**	—	—	**1.9234**	**0.0109**		
10555438	NM_199012	**Fchsd2**	FCH and double SH3 domains 2	7 E3	**1.9147**	**0.0156**		
10436100	NM_181596	**Retnlg**	resistin like gamma	16 B5|16 32.5 cM	**1.9126**	**0.0028**		
10542594	AY512955	**Gm10210**	predicted gene 10210	—	**1.9107**	**0.047**		
10554693	NM_023377	**Stard5**	StAR-related lipid transfer (START) domain containing 5	7 D3	**1.903**	**0.0204**		
10391744	AK135410	**Gpatch8**	G patch domain containing 8	11 E1	**1.9025**	**0.0148**		
10560624	NM_009696	**Apoe**	apolipoprotein E	7 A3|7 4.0 cM	**1.8965**	**0.0301**		
10542880	BC048711	**4833442J19Rik**	RIKEN cDNA 4833442J19 gene	6 G3	**1.8917**	**0.0144**		
10461150	—	**—**	—	—	**1.8816**	**0.0397**		
10585982	NM_173018	**Myo9a**	myosin IXa	9 B	**1.8764**	**0.019**		
10547469	NM_001037155	**Hsn2**	hereditary sensory neuropathy, type II	6 F1	**1.8743**	**0.0152**		
10482762	NM_145360	**Idi1**	isopentenyl-diphosphate delta isomerase	13 A1	**1.8722**	**0.0143**		
10411147	NM_022884	**Bhmt2**	betaine-homocysteine methyltransferase 2	13 C3|13 49.0 cM	**1.8637**	**0.0317**		
10364038	NM_133995	**Upb1**	ureidopropionase, beta	10 C1	**1.8607**	**0.0099**		
10382888	AK171830	**2810008D09Rik**	RIKEN cDNA 2810008D09 gene	—	**1.8559**	**0.0272**		
10485170	NM_009963	**Cry2**	cryptochrome 2 (photolyase-like)	2 E	**1.8553**	**0.0324**		
10594988	NM_015806	**Mapk6**	mitogen-activated protein kinase 6	9 D|9 38.0 cM	**1.8546**	**0.0327**		
10535938	NM_133898	**N4bp2l1**	NEDD4 binding protein 2-like 1	5 G3	**1.8517**	**0.0189**		
10556769	NM_016870	**Acsm3**	acyl-CoA synthetase medium-chain family member 3	7 F3	**1.8511**	**0.0207**		
10401149	NM_013738	**Plek2**	pleckstrin 2	12 D2	**1.8429**	**0.0098**		
10599192	NM_028894	**Lonrf3**	LON peptidase N-terminal domain and ring finger 3	X A2	**1.8374**	**0.035**		
10391746	NM_001159492	**Gpatch8**	G patch domain containing 8	11 E1	**1.8331**	**0.0248**		
10403413	NM_145360	**Idi1**	isopentenyl-diphosphate delta isomerase	13 A1	**1.8228**	**0.0272**		
10580955	—	**—**	—	—	**1.8221**	**0.0277**		
10396730	—	**—**	—	—	**1.8218**	**0.0374**		
10508800	AY140896	**Gm3579**	predicted gene 3579	4 D2.3|4	**1.8214**	**0.0197**		
10573865	AY140896	**Gm3579**	predicted gene 3579	4 D2.3|4	**1.8214**	**0.0197**		
10558961	NM_053082	**Tspan4**	tetraspanin 4	7 F5	**1.8183**	**0.0132**		
10350753	NM_008131	**Glul**	glutamate-ammonia ligase (glutamine synthetase)	—	**1.817**	**0.0285**		
10351043	NR_028543	**Snord47**	small nucleolar RNA, C/D box 47	1|1	**1.8156**	**0.0327**		
10377439	NM_001159367	**Per1**	period homolog 1 (Drosophila)	11 B	**1.8092**	**0.0359**		
10520388	NM_028234	**Rbm33**	RNA binding motif protein 33	5 B1	**1.8056**	**0.0379**		
10370931	NM_021462	**Mknk2**	MAP kinase-interacting serine/threonine kinase 2	10 C1	**1.7989**	**0.0023**		
10582658	NM_007428	**Agt**	angiotensinogen (serpin peptidase inhibitor, clade A, member 8)	8 E2|8 68.0 cM	**1.7834**	**0.0217**		
10472860	NM_019688	**Rapgef4**	Rap guanine nucleotide exchange factor (GEF) 4	2 C3	**1.7824**	**0.0118**		
10505489	NM_021362	**Pappa**	pregnancy-associated plasma protein A	4 C1|4 32.2 cM	**1.7809**	**0.0012**		
10377751	NM_009714	**Asgr1**	asialoglycoprotein receptor 1	11 B3|11 37.0 cM	**1.7793**	**0.0146**		
10351224	NM_007976	**F5**	coagulation factor V	1 H2.2|1 86.6 cM	**1.7735**	**0.0277**		
10372988	NM_011391	**Slc16a7**	solute carrier family 16 (monocarboxylic acid transporters), member 7	10 D3	**1.7729**	**0.0311**		
10351259	NM_054087	**Slc19a2**	solute carrier family 19 (thiamine transporter), member 2	1 H2.2|1 87.0 cM	**1.7698**	**0.0242**		
10409876	NM_007796	**Ctla2a**	cytotoxic T lymphocyte-associated protein 2 alpha	13 B2|13 36.0 cM	**1.7598**	**0.0424**		
10410877	NM_001081176	**Polr3g**	polymerase (RNA) III (DNA directed) polypeptide G	13 C3	**1.7388**	**0.0345**		
10424686	BC025446	**BC025446**	cDNA sequence BC025446	15 D3	**1.7378**	**0.0152**		
10351533	NM_009803	**Nr1i3**	nuclear receptor subfamily 1, group I, member 3	1 H3|1 92.6 cM	**1.7285**	**0.0341**		
10533050	NM_030704	**Hspb8**	heat shock protein 8	5 F|5 59.0 cM	**1.716**	**0.0394**		
10496872	NM_133222	**Eltd1**	EGF, latrophilin seven transmembrane domain containing 1	3 H3-H4	**1.7157**	**0.0233**		
10518743	NM_173371	**H6pd**	hexose-6-phosphate dehydrogenase (glucose 1-dehydrogenase)	4 E2|4 78.4 cM	**1.7094**	**0.0165**		
10487021	NM_011774	**Slc30a4**	solute carrier family 30 (zinc transporter), member 4	2 E5|2 69.0 cM	**1.7066**	**0.0161**		
10595622	—	**—**	—	—	**1.7056**	**0.0342**		
10595636	—	**—**	—	—	**1.7056**	**0.0342**		
10595626	—	**—**	—	—	**1.7034**	**0.029**		
10446425	—	**—**	—	—	**1.7003**	**0.0214**		
10473160	NM_080558	**Ssfa2**	sperm specific antigen 2	2 D	**1.6908**	**0.0159**		
10375229	—	**—**	—	—	**1.6902**	**0.0112**		
10592938	NM_138951	**Ttc36**	tetratricopeptide repeat domain 36	9 A5.2	**1.6901**	**0.04**		
10394699	NM_009072	**Rock2**	Rho-associated coiled-coil containing protein kinase 2	12 A3	**1.6898**	**0.0376**		
10354085	NM_019570	**Rev1**	REV1 homolog (S. cerevisiae)	1 B	**1.6849**	**0.0097**		
10462922	NM_019588	**Plce1**	phospholipase C, epsilon 1	19 D1	**1.6829**	**0.032**		
10395259	NM_021524	**Nampt**	nicotinamide phosphoribosyltransferase	12 B1	**1.676**	**0.0284**		
10597875	NM_010012	**Cyp8b1**	cytochrome P450, family 8, subfamily b, polypeptide 1	9 F4|9 71.0 cM	**1.6755**	**0.013**		
10344809	NM_026493	**Cspp1**	centrosome and spindle pole associated protein 1	1 A2	**1.6735**	**0.0484**		
10347748	NM_028817	**Acsl3**	acyl-CoA synthetase long-chain family member 3	1 C4|1 24.1 cM	**1.6733**	**0.019**		
10375216	NM_145962	**Pank3**	pantothenate kinase 3	11 A4	**1.6733**	**0.013**		
10545877	NM_023455	**Nat8**	N-acetyltransferase 8 (GCN5-related, putative)	6 C3	**1.6673**	**0.0378**		
10374453	NM_008131	**Glul**	glutamate-ammonia ligase (glutamine synthetase)	—	**1.666**	**0.0345**		
10515690	NM_198170	**Szt2**	seizure threshold 2	4 D2.1	**1.6601**	**0.0207**		
10591612	NM_177030	**Dock6**	dedicator of cytokinesis 6	9 A3	**1.6594**	**0.0225**		
10389214	NM_011338	**Ccl9**	chemokine (C-C motif) ligand 9	11 C|11 47.4 cM	**1.6564**	**0.0355**		
10587688	—	**—**	—	—	**1.6554**	**0.0277**		
10595620	—	**—**	—	—	**1.6554**	**0.0277**		
10366275	—	**—**	—	—	**1.6481**	**0.0095**		
10607089	NM_207625	**Acsl4**	acyl-CoA synthetase long-chain family member 4	X F2	**1.647**	**0.0484**		
10379820	NM_133360	**Acaca**	acetyl-Coenzyme A carboxylase alpha	11 C	**1.6452**	**0.0014**		
10414537	NM_001161731	**Ang**	angiogenin, ribonuclease, RNase A family, 5	14 B-C1|14 18.0 cM	**1.6406**	**0.0094**		
10505779	NM_139306	**Acer2**	alkaline ceramidase 2	4 C4	**1.6403**	**0.0141**		
10564183	AF241256	**Snord116**	small nucleolar RNA, C/D box 116 cluster	7 C|7 29.0 cM	**1.6393**	**0.0126**		
10544573	NM_027852	**Rarres2**	retinoic acid receptor responder (tazarotene induced) 2	6 B2.3	**1.6174**	**0.0361**		
10472436	NM_020283	**B3galt1**	UDP-Gal:betaGlcNAc beta 1,3-galactosyltransferase, polypeptide 1	2 C3	**1.6168**	**0.0376**		
10515399	NM_013807	**Plk3**	polo-like kinase 3 (Drosophila)	4 D1	**1.616**	**0.039**		
10407876	NM_138654	**5033411D12Rik**	RIKEN cDNA 5033411D12 gene	13 A2	**1.6108**	**0.0317**		
10478847	NM_001081005	**1500012F01Rik**	RIKEN cDNA 1500012F01 gene	2 H3	**1.6077**	**0.0445**		
10362896	NM_009846	**Cd24a**	CD24a antigen	10 B2|10 26.0 cM	**1.6059**	**0.0272**		
10535956	NM_001163493	**Stard13**	StAR-related lipid transfer (START) domain containing 13	5 G3	**1.6025**	**0.0225**		
10371400	NM_007771	**Cry1**	cryptochrome 1 (photolyase-like)	10 C|10 46.0 cM	**1.5989**	**0.0109**		
10540122	NM_009320	**Slc6a6**	solute carrier family 6 (neurotransmitter transporter, taurine), member 6	6 D1|6 38.2 cM	**1.5937**	**0.0136**		
10580953	—	**—**	—	—	**1.5913**	**0.0232**		
10356082	NM_011636	**Plscr1**	phospholipid scramblase 1	9 E3.3	**1.5899**	**0.036**		
10453272	NM_001001806	**Zfp36l2**	zinc finger protein 36, C3H type-like 2	17 E4	**1.5897**	**0.013**		
10488322	NM_001033348	**Ralgapa2**	Ral GTPase activating protein, alpha subunit 2 (catalytic)	2 G1	**1.5885**	**0.0019**		
10462406	NM_001081319	**C030046E11Rik**	RIKEN cDNA C030046E11 gene	19 C1	**1.5858**	**0.0471**		
10382797	NM_176902	**Fam100b**	family with sequence similarity 100, member B	11 E2	**1.583**	**0.023**		
10346328	XR_031547	**Gm8292**	predicted gene 8292	1 C1.2	**1.5828**	**0.0066**		
10459766	NR_028560	**Scarna17**	small Cajal body-specific RNA 17	18|18	**1.5813**	**0.0232**		
10461642	NR_028560	**Scarna17**	small Cajal body-specific RNA 17	18|18	**1.5813**	**0.0232**		
10474545	NM_133649	**Slc12a6**	solute carrier family 12, member 6	2 E3	**1.5812**	**0.0361**		
10471503	BC110660	**Taf1d**	TATA box binding protein (Tbp)-associated factor, RNA polymerase I, D	9 A3	**1.5797**	**0.0352**		
10572865	NM_027837	**Isx**	intestine specific homeobox	8 C2	**1.5776**	**0.0224**		
10357345	NM_172484	**Nckap5**	NCK-associated protein 5	1 E3	**1.5726**	**0.0374**		
10501494	NM_001042711	**Amy2a5**	amylase 2a5	3 F3|3 50.0 cM	**1.5715**	**0.015**		
10558295	ENSMUST00000106157	**Zranb1**	zinc finger, RAN-binding domain containing 1	7 F3	**1.5699**	**0.0362**		
10553829	NR_003376	**Gm7367**	1110014K08Rik pseudogene	7 C|7	**1.5663**	**0.0376**		
10553831	NR_003376	**Gm7367**	1110014K08Rik pseudogene	7 C|7	**1.5663**	**0.0376**		
10557233	NM_144925	**Tnrc6a**	trinucleotide repeat containing 6a	7 F3	**1.5644**	**0.0447**		
10439667	NM_145389	**BC016579**	cDNA sequence, BC016579	16 B5	**1.5633**	**0.0355**		
10354168	NM_018775	**Tbc1d8**	TBC1 domain family, member 8	1 B	**1.5603**	**0.0205**		
10418169	ENSMUST00000079800 // ENSMUST00000079800	**1700054O19Rik // 1700054O19Rik**	RIKEN cDNA 1700054O19 gene // RIKEN cDNA 1700054O19 gene	14 A3 // 14 A3	**1.5539**	**0.0023**		
10509063	NM_178257	**Il22ra1**	interleukin 22 receptor, alpha 1	4 D3|4 62.0 cM	**1.5466**	**0.0232**		
10391732	NM_001159492	**Gpatch8**	G patch domain containing 8	11 E1	**1.5421**	**0.0168**		
10399198	NM_019744	**Ncoa4**	nuclear receptor coactivator 4	14 B	**1.5412**	**0.0444**		
10512774	NM_178893	**Coro2a**	coronin, actin binding protein 2A	4 B1	**1.5393**	**0.0307**		
10485633	ENSMUST00000099651	**Gm10796**	predicted gene 10796	—	**1.5366**	**0.0286**		
10350758	AK080751	**A930039A15Rik**	RIKEN cDNA A930039A15 gene	—	**1.5361**	**0.044**		
10427461	NM_001136079	**Ptger4**	prostaglandin E receptor 4 (subtype EP4)	15 A1|15 6.4 cM	**1.5341**	**0.0233**		
10417887	NM_029104	**Zmynd17**	zinc finger, MYND domain containing 17	14 B	**1.531**	**0.0494**		
10436209	NM_001033238	**Cblb**	Casitas B-lineage lymphoma b	16 B5	**1.5287**	**0.0345**		
10587792	NM_011636	**Plscr1**	phospholipid scramblase 1	9 E3.3	**1.5275**	**0.0242**		
10469300	NM_177268	**Ankrd16**	ankyrin repeat domain 16	2 A1	**1.5231**	**0.0313**		
10497487	—	**—**	—	—	**1.5217**	**0.0217**		
10510286	NM_027985	**Mad2l2**	MAD2 mitotic arrest deficient-like 2 (yeast)	4 E1	**1.52**	**0.0152**		
10475866	NM_207680	**Bcl2l11**	BCL2-like 11 (apoptosis facilitator)	2 F3-G1	**1.5187**	**0.0194**		
10497421	NM_080634	**Hps3**	Hermansky-Pudlak syndrome 3 homolog (human)	3 A2|3 12.5 cM	**1.5153**	**0.0272**		
10538356	NM_001163640	**Chn2**	chimerin (chimaerin) 2	6 B3	**1.5142**	**0.0215**		
10475946	NM_178404	**Zc3h6**	zinc finger CCCH type containing 6	2 F1	**1.5139**	**0.0465**		
10584350	NM_009429	**Tpt1**	tumor protein, translationally-controlled 1	14 D3	**1.5134**	**0.0362**		
10523856	—	**—**	—	—	**1.5131**	**0.0416**		
10564159	AF241256	**Snord116**	small nucleolar RNA, C/D box 116 cluster	7 C|7 29.0 cM	**1.5126**	**0.0148**		
10559916	—	**—**	—	—	**1.5073**	**0.0309**		
10410513	NM_001040692	**Slc6a18**	solute carrier family 6 (neurotransmitter transporter), member 18	13 C1|13 42.0 cM	**1.506**	**0.0469**		
10413839	NM_001033988	**Ncoa4**	nuclear receptor coactivator 4	14 B	**1.5054**	**0.048**		
10459768	—	**—**	—	—	**1.5005**	**0.0316**		
10562234	NM_001110252	**Hpn**	hepsin	7 B1	**1.4972**	**0.0454**		
10407370	NM_001113550	**4833420G17Rik**	RIKEN cDNA 4833420G17 gene	13 D2.3	**1.4954**	**0.0245**		
10517703	—	**—**	—	—	**1.4948**	**0.0146**		
10497441	NM_013755	**Gyg**	glycogenin	3 A2|3 12.5 cM	**1.4893**	**0.0056**		
10470959	NM_172267	**Phyhd1**	phytanoyl-CoA dioxygenase domain containing 1	2 B	**1.4891**	**0.0376**		
10601755	NM_019865	**Rpl36a**	ribosomal protein L36A	X E3	**1.4875**	**0.0384**		
10355050	NM_001045513	**Raph1**	Ras association (RalGDS/AF-6) and pleckstrin homology domains 1	1 C2	**1.4843**	**0.0308**		
10514500	NM_001004141	**Cyp2j11**	cytochrome P450, family 2, subfamily j, polypeptide 11	4 C5	**1.483**	**0.045**		
10497399	NM_001122759	**Pde7a**	phosphodiesterase 7A	3 A2|3 7.0 cM	**1.4697**	**0.0106**		
10532040	NM_026856	**Zfp644**	zinc finger protein 644	5 E5|5 56.0 cM	**1.4674**	**0.0484**		
10421972	NM_019865	**Rpl36a**	ribosomal protein L36A	X E3	**1.467**	**0.0489**		
10399478	NM_015763	**Lpin1**	lipin 1	12 A1.1|12 9.0 cM	**1.4665**	**0.0214**		
10438358	NM_213614	**5-Sep**	septin 5	16 A3|16 11.42 cM	**1.4654**	**0.0468**		
10544219	NM_139294	**Braf**	Braf transforming gene	6 B1|6 15.5 cM	**1.4639**	**0.0078**		
10358894	NM_146126	**Sord**	sorbitol dehydrogenase	2 E5|2 66.0 cM	**1.4618**	**0.0277**		
10401997	NM_011877	**Ptpn21**	protein tyrosine phosphatase, non-receptor type 21	12 F1	**1.4616**	**0.0405**		
10475437	NM_146126	**Sord**	sorbitol dehydrogenase	2 E5|2 66.0 cM	**1.4599**	**0.0325**		
10379034	NM_026708	**Tlcd1**	TLC domain containing 1	11 B5	**1.4516**	**0.0339**		
10497935	—	**—**	—	—	**1.4454**	**0.0361**		
10389025	NM_177390	**Myo1d**	myosin ID	11 B5|11 46.0 cM	**1.443**	**0.0454**		
10389022	NM_177390	**Myo1d**	myosin ID	11 B5|11 46.0 cM	**1.4428**	**0.0447**		
10396956	NM_018814	**Pcnx**	pecanex homolog (Drosophila)	12 D1	**1.4386**	**0.0122**		
10462346	NM_021525	**Rcl1**	RNA terminal phosphate cyclase-like 1	19 C1	**1.4359**	**0.0151**		
10352416	NM_130890	**Capn8**	calpain 8	1 H4	**1.4317**	**0.0233**		
10592001	NM_011176	**St14**	suppression of tumorigenicity 14 (colon carcinoma)	9 A4|9 17.0 cM	**1.4289**	**0.0123**		
10507110	—	**—**	—	—	**1.4264**	**0.0448**		
10552030	NM_008085	**Gapdhs**	glyceraldehyde-3-phosphate dehydrogenase, spermatogenic	7 B1	**1.4162**	**0.0437**		
10447429	ENSMUST00000072072	**Gm4832**	predicted gene 4832	17 E4	**1.4137**	**0.035**		
10520371	NM_028234	**Rbm33**	RNA binding motif protein 33	5 B1	**1.4117**	**0.045**		
10402117	NM_153587	**Rps6ka5**	ribosomal protein S6 kinase, polypeptide 5	12 E	**1.4072**	**0.048**		
10481827	NM_001085507	**Zbtb34**	zinc finger and BTB domain containing 34	2 B	**1.4071**	**0.0194**		
10526656	NM_146164	**Lrch4**	leucine-rich repeats and calponin homology (CH) domain containing 4	5 G2	**1.404**	**0.0447**		
10582811	NM_001164598	**Irf2bp2**	interferon regulatory factor 2 binding protein 2	8 E2	**1.3943**	**0.0286**		
10493343	—	**—**	—	—	**1.3926**	**0.0441**		
10474725	NM_013719	**Eif2ak4**	eukaryotic translation initiation factor 2 alpha kinase 4	—	**1.3874**	**0.0301**		
10489377	NM_012032	**Serinc3**	serine incorporator 3	2 H3	**1.3784**	**0.0274**		
10480570	NM_001162485	**Arrdc1**	arrestin domain containing 1	2 A3	**1.3734**	**0.0478**		
10366446	NM_146010	**Tspan8**	tetraspanin 8	10 D2	**1.3733**	**0.0324**		
10346970	NM_011086	**Pikfyve**	phosphoinositide kinase, FYVE finger containing	1 C2	**1.3711**	**0.0175**		
10384423	NM_172496	**Cobl**	cordon-bleu	11 A1|11 6.5 cM	**1.3708**	**0.048**		
10476443	NM_013829	**Plcb4**	phospholipase C, beta 4	2 F3|2 77.0 cM	**1.3705**	**0.0333**		
10534889	NM_178162	**Agfg2**	ArfGAP with FG repeats 2	5 G2	**1.369**	**0.0181**		
10518428	NM_011929	**Clcn6**	chloride channel 6	4 76.4 cM	**1.3681**	**0.0262**		
10542264	NM_001163445	**2700089E24Rik**	RIKEN cDNA 2700089E24 gene	6 G1	**1.3677**	**0.045**		
10497485	—	**—**	—	—	**1.3662**	**0.0333**		
10349065	NM_001122676	**Zcchc2**	zinc finger, CCHC domain containing 2	1 E2.1	**1.3603**	**0.0286**		
10477311	NM_001039939	**Asxl1**	additional sex combs like 1 (Drosophila)	2 H1	**1.3592**	**0.0395**		
10432122	NM_001170711	**Asb8**	ankyrin repeat and SOCS box-containing 8	15 F2	**1.3504**	**0.0362**		
10567229	NM_001031814	**Smg1**	SMG1 homolog, phosphatidylinositol 3-kinase-related kinase (C. elegans)	7 F2	**1.3481**	**0.0485**		
10376312	NM_028451	**Larp1**	La ribonucleoprotein domain family, member 1	11 B2	**1.3375**	**0.0311**		
10503161	NM_001081417	**Chd7**	chromodomain helicase DNA binding protein 7	4 A1|4 1.0 cM	**1.3312**	**0.0407**		
10587150	NM_001081322	**Myo5c**	myosin VC	9 D	**1.3133**	**0.0489**		
10476033	NM_183262	**Stk35**	serine/threonine kinase 35	2 F1	**1.2947**	**0.0412**		
10466530	NM_001163144	**Pcsk5**	proprotein convertase subtilisin/kexin type 5	19 B|19 18.0 cM	**1.2894**	**0.0362**		
10478219	NM_021280	**Plcg1**	phospholipase C, gamma 1	2 H2|2 92.0 cM	**1.2708**	**0.0381**		
10367919	NM_029075	**Stx11**	syntaxin 11	10 A1	**−1.2794**	**0.048**		
10605319	NM_145405	**Ubl4**	ubiquitin-like 4	X A7.3|X 29.9 cM	**−1.2895**	**0.048**		
10508089	NM_025544	**Mrps15**	mitochondrial ribosomal protein S15	4 D2.1	**−1.2959**	**0.0479**		
10591482	NM_016679	**Keap1**	kelch-like ECH-associated protein 1	9 A3	**−1.298**	**0.0397**		
10591739	NM_001102404	**Acp5**	acid phosphatase 5, tartrate resistant	9 A3|9 6.0 cM	**−1.3007**	**0.0272**		
10551159	XR_034166	**Gm4607**	predicted gene 4607	7 A3|7	**−1.3016**	**0.0479**		
10413222	NM_134084	**Ppif**	peptidylprolyl isomerase F (cyclophilin F)	14 A3	**−1.3037**	**0.048**		
10356657	NM_024197	**Ndufa10**	NADH dehydrogenase (ubiquinone) 1 alpha subcomplex 10	1 D	**−1.3055**	**0.0479**		
10347672	NM_027886	**Stk11ip**	serine/threonine kinase 11 interacting protein	1 C3	**−1.3059**	**0.0438**		
10439695	NM_019754	**Tagln3**	transgelin 3	16 B5	**−1.3087**	**0.0387**		
10593591	NM_144784	**Acat1**	acetyl-Coenzyme A acetyltransferase 1	9 30.0 cM	**−1.3115**	**0.0418**		
10432866	NM_001003667	**Krt77**	keratin 77	15 F3	**−1.3141**	**0.0484**		
10378443	NM_001011851	**Olfr412**	olfactory receptor 412	11 B5	**−1.3165**	**0.048**		
10487629	NM_130884	**Idh3b**	isocitrate dehydrogenase 3 (NAD+) beta	2 F3	**−1.3214**	**0.0265**		
10432636	NM_174992	**Smagp**	small cell adhesion glycoprotein	15 F1	**−1.3221**	**0.0376**		
10543684	NR_030713	**Mir183**	microRNA 183	—	**−1.3247**	**0.0355**		
10411156	NM_029153	**Scamp1**	secretory carrier membrane protein 1	13 D1	**−1.3284**	**0.036**		
10585417	NM_029573	**Idh3a**	isocitrate dehydrogenase 3 (NAD+) alpha	9 A5.3	**−1.3357**	**0.0367**		
10415742	NM_027436	**Mipep**	mitochondrial intermediate peptidase	14 D1	**−1.3368**	**0.0307**		
10406205	NM_030711	**Erap1**	endoplasmic reticulum aminopeptidase 1	13 C1	**−1.3442**	**0.0454**		
10449940	NM_022434	**Cyp4f14**	cytochrome P450, family 4, subfamily f, polypeptide 14	17 B1	**−1.3479**	**0.0363**		
10420225	NM_010780	**Cma1**	chymase 1, mast cell	14 C3|14 20.0 cM	**−1.3516**	**0.0307**		
10390685	NM_025661	**Ormdl3**	ORM1-like 3 (S. cerevisiae)	11 D	**−1.3522**	**0.0445**		
10484431	NM_025868	**Tmx2**	thioredoxin-related transmembrane protein 2	2 D	**−1.3536**	**0.0486**		
10515930	BC076608	**AA415398**	expressed sequence AA415398	4 D2.1	**−1.3575**	**0.0362**		
10607643	—	**—**	—	—	**−1.3579**	**0.0374**		
10386416	NM_001011789	**Olfr222**	olfactory receptor 222	11 B1.3	**−1.3596**	**0.0447**		
10488291	NM_015754	**Rbbp9**	retinoblastoma binding protein 9	2 G1-H1	**−1.3617**	**0.032**		
10447551	NM_027457	**5730437N04Rik**	RIKEN cDNA 5730437N04 gene	17 A1	**−1.3687**	**0.0396**		
10464128	NM_007611	**Casp7**	caspase 7	19 D2|19 50.0 cM	**−1.3699**	**0.0383**		
10377286	NM_001081566	**Pik3r6**	phosphoinositide-3-kinase, regulatory subunit 6	11 B3	**−1.3731**	**0.0233**		
10539472	NM_019542	**Nagk**	N-acetylglucosamine kinase	6 D1	**−1.3736**	**0.0143**		
10434191	NM_013711	**Txnrd2**	thioredoxin reductase 2	16 A3|16 11.2 cM	**−1.3797**	**0.0324**		
10369525	BC024943	**2010107G23Rik**	RIKEN cDNA 2010107G23 gene	10 B4	**−1.3815**	**0.0461**		
10465826	NM_001160356	**AI462493**	expressed sequence AI462493	19 A	**−1.3869**	**0.0399**		
10435617	NM_001042499	**Rabl3**	RAB, member of RAS oncogene family-like 3	16 B3	**−1.3924**	**0.0387**		
10495285	NM_019972	**Sort1**	sortilin 1	3 F3	**−1.3932**	**0.035**		
10447354	NM_025868	**Tmx2**	thioredoxin-related transmembrane protein 2	2 D	**−1.3953**	**0.0338**		
10575894	NM_026648	**Lrrc50**	leucine rich repeat containing 50	8 E1	**−1.3988**	**0.0418**		
10368893	NM_145743	**Lace1**	lactation elevated 1	10 B2|10 25.5 cM	**−1.399**	**0.0265**		
10470050	NM_007379	**Abca2**	ATP-binding cassette, sub-family A (ABC1), member 2	2 A2-B|2 12.6 cM	**−1.4023**	**0.0316**		
10566618	NM_206897	**Olfr6**	olfactory receptor 6	7 E3	**−1.4057**	**0.0416**		
10526514	NM_021719	**Cldn15**	claudin 15	5 G2	**−1.4077**	**0.0418**		
10483679	NM_001080707	**Gpr155**	G protein-coupled receptor 155	2 C3	**−1.4079**	**0.035**		
10476301	NM_001177833	**Smox**	spermine oxidase	2 F1	**−1.408**	**0.0478**		
10365116	NM_133964	**Dohh**	deoxyhypusine hydroxylase/monooxygenase	10 C1	**−1.4203**	**0.0304**		
10386086	NM_153079	**Nmur2**	neuromedin U receptor 2	11 B1.3	**−1.4289**	**0.043**		
10442370	NM_019910	**Dcpp1**	demilune cell and parotid protein 1	17 A3.3|17 10.0 cM	**−1.4357**	**0.043**		
10593169	NM_023114	**Apoc3**	apolipoprotein C-III	9 A5.2|9 27.0 cM	**−1.4359**	**0.0317**		
10579508	NM_001164679	**Ano8**	anoctamin 8	8 B3.3	**−1.4375**	**0.015**		
10431300	NM_001142357	**Alg12**	asparagine-linked glycosylation 12 homolog (yeast, alpha-1,6-mannosyltransferase)	15 E3	**−1.4389**	**0.0334**		
10585874	NM_010421	**Hexa**	hexosaminidase A	9 B|9 29.0 cM	**−1.4394**	**0.0245**		
10571302	NM_026432	**Tmem66**	transmembrane protein 66	8 A4	**−1.4412**	**0.0307**		
10519652	NR_003596	**Gm6455**	predicted gene 6455	5 A1	**−1.4455**	**0.0286**		
10397975	NM_026790	**Ifi27l1**	interferon, alpha-inducible protein 27 like 1	12 E|12 51.0 cM	**−1.4507**	**0.0331**		
10362904	NM_130892	**Rtn4ip1**	reticulon 4 interacting protein 1	10 B2|10 29.0 cM	**−1.4553**	**0.047**		
10352767	NM_010892	**Nek2**	NIMA (never in mitosis gene a)-related expressed kinase 2	1 H6|1 103.0 cM	**−1.4555**	**0.0145**		
10480699	NM_031843	**Dpp7**	dipeptidylpeptidase 7	2 A3	**−1.4566**	**0.0363**		
10529454	ENSMUST00000069741	**E130018O15Rik**	RIKEN cDNA E130018O15 gene	5 B3	**−1.4683**	**0.0429**		
10600531	—	**—**	—	—	**−1.4695**	**0.0218**		
10426812	NM_010271	**Gpd1**	glycerol-3-phosphate dehydrogenase 1 (soluble)	15 56.8 cM	**−1.4746**	**0.0375**		
10519688	NR_003596	**Gm6455**	predicted gene 6455	5 A1	**−1.4804**	**0.0063**		
10518679	NM_133435	**Nmnat1**	nicotinamide nucleotide adenylyltransferase 1	4 E2	**−1.4814**	**0.048**		
10357418	NM_001081078	**Lct**	lactase	1 E4	**−1.4902**	**0.0333**		
10559590	NM_207270	**Ptprh**	protein tyrosine phosphatase, receptor type, H	7 A1	**−1.494**	**0.0286**		
10525365	NM_001042489	**Hvcn1**	hydrogen voltage-gated channel 1	5 F	**−1.4944**	**0.0376**		
10424929	NM_172960	**Adck5**	aarF domain containing kinase 5	15 D3	**−1.4964**	**0.043**		
10351473	NM_001033499	**Sh2d1b2**	SH2 domain protein 1B2	1 H3	**−1.4988**	**0.0061**		
10547976	NM_145391	**Tapbpl**	TAP binding protein-like	6 F3	**−1.5013**	**0.041**		
10521391	NM_030721	**Acox3**	acyl-Coenzyme A oxidase 3, pristanoyl	—	**−1.5034**	**0.0098**		
10490104	NM_011497	**Aurka**	aurora kinase A	2 H3|2 100.0 cM	**−1.5077**	**0.035**		
10411633	NM_008670	**Naip1**	NLR family, apoptosis inhibitory protein 1	13 D1-D3|13 54.0 cM	**−1.5078**	**0.0232**		
10450145	NM_013585	**Psmb9**	proteasome (prosome, macropain) subunit, beta type 9 (large multifunctional peptidase 2)	17 B1|17 18.59 cM	**−1.5167**	**0.045**		
10524681	NM_026933	**Triap1**	TP53 regulated inhibitor of apoptosis 1	5 F	**−1.517**	**0.0058**		
10490491	NM_008093	**Gata5**	GATA binding protein 5	2 H4|2 106.0 cM	**−1.5174**	**0.0361**		
10437655	NM_011955	**Nubp1**	nucleotide binding protein 1	16 A1|16 3.4 cM	**−1.5217**	**0.0461**		
10441530	NM_031395	**Sytl3**	synaptotagmin-like 3	17 A1	**−1.5249**	**0.0443**		
10562130	NM_194057	**Ffar1**	free fatty acid receptor 1	7 B1	**−1.526**	**0.0014**		
10463704	NM_020577	**As3mt**	arsenic (+3 oxidation state) methyltransferase	19 D1	**−1.5357**	**0.0162**		
10524878	NM_001033311	**Vsig10**	V-set and immunoglobulin domain containing 10	5 F	**−1.5404**	**0.0046**		
10393573	NM_011150	**Lgals3bp**	lectin, galactoside-binding, soluble, 3 binding protein	11 E	**−1.5406**	**0.0119**		
10606714	NM_013463	**Gla**	galactosidase, alpha	X E-F1|X 53.0 cM	**−1.5428**	**0.0277**		
10458547	NR_028061	**Gm8615**	glucosamine-6-phosphate deaminase 1 pseudogene	5 G3	**−1.5451**	**0.0246**		
10384378	NM_016672	**Ddc**	dopa decarboxylase	11 A1-A4|11 7.0 cM	**−1.5472**	**0.043**		
10429638	XR_032493	**Gm9568**	glyceraldehyde-3-phosphate dehydrogenase pseudogene	15 D3|15	**−1.5502**	**0.0482**		
10510516	NM_019741	**Slc2a5**	solute carrier family 2 (facilitated glucose transporter), member 5	4 E2	**−1.5555**	**0.0065**		
10565634	NM_008663	**Myo7a**	myosin VIIA	7 E2|7 48.1 cM	**−1.5718**	**0.0053**		
10524422	NM_010018	**Dao**	D-amino acid oxidase	5 F|5 65.0 cM	**−1.575**	**0.0418**		
10566583	AK172683	**Gm8995**	predicted gene 8995	7 E3	**−1.5789**	**0.0371**		
10391207	NM_030150	**Dhx58**	DEXH (Asp-Glu-X-His) box polypeptide 58	11 D|11 61.5 cM	**−1.5797**	**0.0135**		
10408879	NM_001033399	**Gfod1**	glucose-fructose oxidoreductase domain containing 1	13 A4	**−1.5847**	**0.013**		
10505954	NM_013690	**Tek**	endothelial-specific receptor tyrosine kinase	4 C5|4 43.6 cM	**−1.5877**	**0.0422**		
10538082	NM_133764	**Atp6v0e2**	ATPase, H+ transporting, lysosomal V0 subunit E2	6 B3	**−1.5918**	**0.0041**		
10545958	NM_013471	**Anxa4**	annexin A4	6 D1|6 38.0 cM	**−1.596**	**0.0411**		
10560474	NM_177691	**Ppm1n**	protein phosphatase, Mg2+/Mn2+ dependent, 1N (putative)	7 A3	**−1.6066**	**0.0158**		
10577792	NM_031257	**Plekha2**	pleckstrin homology domain-containing, family A (phosphoinositide binding specific) member 2	8 A2	**−1.623**	**0.0343**		
10455784	NM_026240	**Gramd3**	GRAM domain containing 3	18 D2	**−1.6238**	**0.0361**		
10467907	NM_145502	**Erlin1**	ER lipid raft associated 1	19 C3	**−1.6274**	**0.0115**		
10492102	NM_144895	**Spg20**	spastic paraplegia 20, spartin (Troyer syndrome) homolog (human)	3 C	**−1.6317**	**0.0301**		
10377927	NM_027445	**Rnf167**	ring finger protein 167	11 B4	**−1.633**	**0.0209**		
10404439	NM_011452	**Serpinb9b**	serine (or cysteine) peptidase inhibitor, clade B, member 9b	13 A3.3|13 12.5 cM	**−1.6346**	**0.0217**		
10566585	ENSMUST00000098144	**Gm1966**	predicted gene 1966	7 E3	**−1.6387**	**0.0248**		
10587873	—	**—**	—	—	**−1.6446**	**0.037**		
10439068	NM_145932	**Osta**	organic solute transporter alpha	16 B3	**−1.6448**	**0.035**		
10376726	NM_001172112	**Dhrs7b**	dehydrogenase/reductase (SDR family) member 7B	11 B2	**−1.6476**	**0.0062**		
10469951	NM_176834	**Rnf208**	ring finger protein 208	2 A3	**−1.6491**	**0.0225**		
10548817	NM_025806	**Plbd1**	phospholipase B domain containing 1	6 G1	**−1.654**	**0.0163**		
10473356	NM_019949	**Ube2l6**	ubiquitin-conjugating enzyme E2L 6	2 E1	**−1.6544**	**0.0048**		
10501235	NM_026764	**Gstm4**	glutathione S-transferase, mu 4	3 F2.3	**−1.6622**	**0.0309**		
10474825	NM_026412	**D2Ertd750e**	DNA segment, Chr 2, ERATO Doi 750, expressed	2 E5	**−1.6757**	**0.0095**		
10463716	NM_033569	**Cnnm2**	cyclin M2	19 C3|19 47.0 cM	**−1.6797**	**0.0023**		
10604337	—	**—**	—	—	**−1.6798**	**0.0307**		
10595081	NM_012033	**Tinag**	tubulointerstitial nephritis antigen	9 D	**−1.6828**	**0.0065**		
10524621	NM_011854	**Oasl2**	2'-5' oligoadenylate synthetase-like 2	5 F	**−1.687**	**0.0489**		
10444810	ENSMUST00000105041	**H2-Q1**	histocompatibility 2, Q region locus 1	17 B1|17 19.14 cM	**−1.6883**	**0.0032**		
10584334	NM_011734	**Siae**	sialic acid acetylesterase	9 A4|9 19.0 cM	**−1.699**	**0.0032**		
10582985	NM_009807	**Casp1**	caspase 1	9 A1|9 1.0 cM	**−1.6991**	**0.0091**		
10488185	NM_009751	**Bfsp1**	beaded filament structural protein 1, in lens-CP94	2 G	**−1.7014**	**0.0096**		
10491091	NM_009425	**Tnfsf10**	tumor necrosis factor (ligand) superfamily, member 10	3 A3	**−1.7131**	**0.0053**		
10583920	NM_026189	**Eepd1**	endonuclease/exonuclease/phosphatase family domain containing 1	9 A4	**−1.7418**	**0.0179**		
10378572	NM_027249	**Tlcd2**	TLC domain containing 2	11 B5	**−1.7499**	**0.0309**		
10383192	—	**—**	—	—	**−1.7677**	**0.0205**		
10559606	NM_023440	**Tmem86b**	transmembrane protein 86B	7 A1	**−1.776**	**0.008**		
10550740	NM_027839	**Ceacam20**	carcinoembryonic antigen-related cell adhesion molecule 20	7 A3	**−1.7837**	**0.0219**		
10486172	NM_001033136	**Fam82a2**	family with sequence similarity 82, member A2	2 E5	**−1.7911**	**0.0045**		
10482802	NM_139200	**Cytip**	cytohesin 1 interacting protein	2 C1.1	**−1.798**	**0.0078**		
10402347	NM_029803	**Ifi27l2a**	interferon, alpha-inducible protein 27 like 2A	12 E	**−1.807**	**0.0173**		
10519196	NM_147776	**Vwa1**	von Willebrand factor A domain containing 1	—	**−1.8075**	**0.0079**		
10488636	NM_001039120	**Defb26**	defensin beta 26	2 H1	**−1.8103**	**0.041**		
10542857	NM_178797	**Far2**	fatty acyl CoA reductase 2	6 G3	**−1.8273**	**0.0308**		
10376832	NM_007413	**Adora2b**	adenosine A2b receptor	11 B2	**−1.8282**	**0.0194**		
10558265	NM_029609	**Lhpp**	phospholysine phosphohistidine inorganic pyrophosphate phosphatase	7 F4	**−1.8296**	**0.001**		
10569203	NM_001142681	**Chid1**	chitinase domain containing 1	7 F5	**−1.8468**	**0.0112**		
10571984	NM_001081215	**Ddx60**	DEAD (Asp-Glu-Ala-Asp) box polypeptide 60	8 B3.1	**−1.8805**	**0.05**		
10424221	NM_001081396	**Wdr67**	WD repeat domain 67	15 D1	**−1.8879**	**0.0054**		
10364134	NM_133184	**Slc5a4a**	solute carrier family 5, member 4a	10 C1	**−1.9264**	**0.0251**		
10447904	NM_199252	**Unc93a**	unc-93 homolog A (C. elegans)	17 A1|17 7.8 cM	**−1.9295**	**0.025**		
10539186	NM_011259	**Reg3a**	regenerating islet-derived 3 alpha	6 C3|6 33.5 cM	**−1.9355**	**0.0286**		
10451287	NM_015783	**Isg15**	ISG15 ubiquitin-like modifier	4 E2|4	**−1.936**	**0.013**		
10447634	NM_001142539	**Gm9992**	predicted gene 9992	17 A1	**−1.9524**	**0.0175**		
10389261	NM_001037932	**Gm11437**	predicted gene 11437	11 C	**−1.9832**	**0.0076**		
10538590	NM_025992	**Herc5**	hect domain and RLD 5	6 C1|6	**−1.9883**	**0.0089**		
10510532	NM_001085529	**Slc2a7**	solute carrier family 2 (facilitated glucose transporter), member 7	4 E2|4	**−2.0053**	**0.0136**		
10385500	NM_008326	**Irgm1**	immunity-related GTPase family M member 1	11 B1.2	**−2.0184**	**0.0234**		
10587871	NM_198414	**Paqr9**	progestin and adipoQ receptor family member IX	9 E3.3	**−2.0207**	**0.0376**		
10568568	NM_016978	**Oat**	ornithine aminotransferase	7 F3|7 63.0 cM	**−2.0471**	**3.00E−04**		
10467470	NM_019698	**Aldh18a1**	aldehyde dehydrogenase 18 family, member A1	19 C3	**−2.3775**	**0.0079**		
10425287	NM_134090	**Kdelr3**	KDEL (Lys-Asp-Glu-Leu) endoplasmic reticulum protein retention receptor 3	15 E1	**−2.4335**	**0.0115**		
10376324	NM_001135115	**Gm12250**	predicted gene 12250	11 B1.3	**−2.4477**	**0.0311**		
10443869	NM_024442	**Cyp4f16**	cytochrome P450, family 4, subfamily f, polypeptide 16	17 B1	**−2.5218**	**0.0265**		
10566571	NR_030719	**Gm8979**	very large inducible GTPase 1 pseudogene	7 E3	**−2.6198**	**0.0145**		
10390748	NM_172564	**Tns4**	tensin 4	11 D	**−2.6277**	**0.0044**		
10462618	NM_010501	**Ifit3**	interferon-induced protein with tetratricopeptide repeats 3	19 C3	**−2.6278**	**0.0357**		
10545200	ENSMUST00000101325	**LOC100046894**	similar to Igk-C protein	—	**−2.6477**	**0.0473**		
10499899	NM_009264	**Sprr1a**	small proline-rich protein 1A	3 F1|3 45.2 cM	**−2.7972**	**0.0286**		
10483074	NM_008100	**Gcg**	glucagon	2 C1.3|2 36.0 cM	**−2.7977**	**0.0019**		
10557300	NM_007474	**Aqp8**	aquaporin 8	7 F3|7 61.0 cM	**−2.7985**	**8.00E−04**		
10566578	NR_030719	**Gm8979**	very large inducible GTPase 1 pseudogene	7 E3	**−2.8066**	**0.0095**		
10430006	NM_028064	**Slc39a4**	solute carrier family 39 (zinc transporter), member 4	15 D3	**−2.8188**	**0.0145**		
10390691	NM_145434	**Nr1d1**	nuclear receptor subfamily 1, group D, member 1	11 D	**−3.298**	**3.00E−04**		
10480633	NM_080854	**Slc34a3**	solute carrier family 34 (sodium phosphate), member 3	2 A3	**−3.3614**	**0.013**		
10566366	NM_199146	**AI451617**	expressed sequence AI451617	7 E3	**−3.9279**	**0.0019**		
10505451	NM_011016	**Orm2**	orosomucoid 2	4 C1|4 31.4 cM			**3.9755**	**0.0307**
10416057	NM_013492	**Clu**	clusterin	14 D1|14 28.0 cM			**3.6483**	**0.0393**
10452854	NM_053188	**Srd5a2**	steroid 5 alpha-reductase 2	17 E2			**3.4167**	**7.00E−04**
10367045	NM_009040	**Rdh16**	retinol dehydrogenase 16	10 D3			**2.3984**	**0.0273**
10523717	NM_009263	**Spp1**	secreted phosphoprotein 1	5 E5|5 56.0 cM			**2.2985**	**0.0406**
10464594	BC034269	**BC021614**	cDNA sequence BC021614	19 A			**2.2581**	**0.011**
10429140	NM_008681	**Ndrg1**	N-myc downstream regulated gene 1	15 D2			**2.087**	**0.0273**
10469609	NR_033225	**Gm13375**	predicted gene 13375	2 A3			**2.0048**	**0.0294**
10507671	NM_008190	**Guca2a**	guanylate cyclase activator 2a (guanylin)	4 D2.1|4 57.0 cM			**1.8922**	**0.0482**
10556113	NM_016809	**Rbm3**	RNA binding motif protein 3	X A1.1|X 2.0 cM			**1.8115**	**0.0271**
10359917	NM_010476	**Hsd17b7**	hydroxysteroid (17-beta) dehydrogenase 7	1 H3			**1.7626**	**0.0467**
10358454	NM_001166409	**Rbm3**	RNA binding motif protein 3	X A1.1|X 2.0 cM			**1.7444**	**0.0294**
10540034	NM_027406	**Aldh1l1**	aldehyde dehydrogenase 1 family, member L1	6 D1			**1.7157**	**0.015**
10371784	NM_001163700	**Nr1h4**	nuclear receptor subfamily 1, group H, member 4	10 C2|10 50.0 cM			**1.7001**	**0.0435**
10603469	NM_001166409	**Rbm3**	RNA binding motif protein 3	X A1.1|X 2.0 cM			**1.6579**	**0.0273**
10582310	NM_138656	**Mvd**	mevalonate (diphospho) decarboxylase	8 E1			**1.6179**	**0.0287**
10518568	—	**—**	—	—			**1.5432**	**0.0336**
10443898	NM_134127	**Cyp4f15**	cytochrome P450, family 4, subfamily f, polypeptide 15	17 B1			**1.5311**	**0.0468**
10344713	NM_016661	**Ahcy**	S-adenosylhomocysteine hydrolase	2 H1|2 89.0 cM			**1.5215**	**0.0425**
10488816	NM_016661	**Ahcy**	S-adenosylhomocysteine hydrolase	2 H1|2 89.0 cM			**1.5127**	**0.0377**
10439762	NM_016661	**Ahcy**	S-adenosylhomocysteine hydrolase	2 H1|2 89.0 cM			**1.5089**	**0.0468**
10605055	NM_028633	**Haus7**	HAUS augmin-like complex, subunit 7	X A7.3			**1.5083**	**0.015**
10556169	NM_025344	**Eif3f**	eukaryotic translation initiation factor 3, subunit F	7 E3			**1.418**	**0.0317**
10413542	NM_009388	**Tkt**	transketolase	14 B1			**1.4043**	**0.0425**
10408850	NM_001111324	**Nedd9**	neural precursor cell expressed, developmentally down-regulated gene 9	13 A3.3-A4			**−1.3853**	**0.0463**
10489107	NM_018851	**Samhd1**	SAM domain and HD domain, 1	2 H2			**−1.4139**	**0.0456**
10516620	NM_010693	**Lck**	lymphocyte protein tyrosine kinase	4 D2.2|4 59.0 cM			**−1.4259**	**0.0348**
10513256	NM_010336	**Lpar1**	lysophosphatidic acid receptor 1	4 B3|4 16.0 cM			**−1.434**	**0.0294**
10604057	NR_033443	**6-Sep**	septin 6	X A2			**−1.4532**	**0.0468**
10588464	NR_029526	**Mirlet7g**	microRNA let7g	—			**−1.464**	**0.0301**
10432362	NM_026967	**Rhebl1**	Ras homolog enriched in brain like 1	15 F2			**−1.49**	**0.017**
10350173	NM_001130174	**Tnnt2**	troponin T2, cardiac	1 E4|1 60.0 cM			**−1.4944**	**0.0425**
10547906	NM_008479	**Lag3**	lymphocyte-activation gene 3	6 F2			**−1.5108**	**0.0346**
10420659	NM_025697	**6330409N04Rik**	RIKEN cDNA 6330409N04 gene	14 D1			**−1.5451**	**0.0354**
10352097	NM_027077	**1700016C15Rik**	RIKEN cDNA 1700016C15 gene	1 H3			**−1.5503**	**0.0468**
10409579	NM_019568	**Cxcl14**	chemokine (C-X-C motif) ligand 14	13 B1			**−1.5799**	**0.0377**
10355960	NM_009129	**Scg2**	secretogranin II	1 C4|1 43.6 cM			**−1.6019**	**0.0178**
10478633	NM_013599	**Mmp9**	matrix metallopeptidase 9	2 H1-H2|2 96.0 cM			**−1.6069**	**0.0228**
10501629	NM_001080818	**Cdc14a**	CDC14 cell division cycle 14 homolog A (S. cerevisiae)	3 G1			**−1.6217**	**0.0425**
10447056	NM_027455	**Qpct**	glutaminyl-peptide cyclotransferase (glutaminyl cyclase)	17 E3			**−1.6326**	**0.0228**
10407940	—	**—**	—	—			**−1.6681**	**0.0294**
10605355	—	**—**	—	—			**−1.6826**	**0.0494**
10593015	NM_009850	**Cd3g**	CD3 antigen, gamma polypeptide	9 A5.2|9 26.0 cM			**−1.6893**	**0.0161**
10464999	NM_028623	**Cst6**	cystatin E/M	19 A|19 4.0 cM			**−1.7219**	**0.0456**
10584821	NM_013487	**Cd3d**	CD3 antigen, delta polypeptide	9 A5.2|9 26.0 cM			**−1.7359**	**0.0435**
10561927	NM_007467	**Aplp1**	amyloid beta (A4) precursor-like protein 1	7 B1|7 8.0 cM			**−1.7367**	**0.0109**
10552406	NM_024253	**Nkg7**	natural killer cell group 7 sequence	7 B2			**−1.7501**	**0.0178**
10366881	NM_007837	**Ddit3**	DNA-damage inducible transcript 3	10 D3			**−1.7621**	**0.0344**
10490923	NM_009801	**Car2**	carbonic anhydrase 2	3 A1|3 10.5 cM			**−1.7726**	**0.0189**
10428534	NM_032000	**Trps1**	trichorhinophalangeal syndrome I (human)	15 C|15 30.1 cM			**−1.793**	**0.0494**
10403821	ENSMUST00000103558	**Tcrg-V3**	T-cell receptor gamma, variable 3	—			**−1.8213**	**0.0468**
10516093	NM_007558	**Bmp8a**	bone morphogenetic protein 8a	4 D2.2|4 57.4 cM			**−1.8392**	**0.0307**
10513739	NM_011607	**Tnc**	tenascin C	4 C1|4 32.2 cM			**−1.853**	**0.0348**
10574166	NM_153507	**Cpne2**	copine II	8 C5			**−1.857**	**0.0346**
10542632	NM_010491	**Iapp**	islet amyloid polypeptide	6 G2|6 62.0 cM			**−1.8827**	**0.0301**
10541605	NM_020001	**Clec4n**	C-type lectin domain family 4, member n	6 F3|6 55.0 cM			**−1.889**	**0.017**
10402325	NM_023049	**Asb2**	ankyrin repeat and SOCS box-containing 2	12 E|12 50.0 cM			**−1.9087**	**0.0492**
10466200	NM_027836	**Ms4a7**	membrane-spanning 4-domains, subfamily A, member 7	19 A			**−1.9359**	**0.0172**
10404913	NM_026056	**Cap2**	CAP, adenylate cyclase-associated protein, 2 (yeast)	13 A5			**−1.9619**	**0.0346**
10420308	NM_013542	**Gzmb**	granzyme B	14 D3|14 20.5 cM			**−1.9705**	**0.0294**
10412211	NM_010370	**Gzma**	granzyme A	13 D|13 64.0 cM			**−2.0182**	**0.0229**
10463016	NM_028191	**Cyp2c65**	cytochrome P450, family 2, subfamily c, polypeptide 65	19 C3			**−2.1252**	**0.0456**
10551197	NM_009999	**Cyp2b10**	cytochrome P450, family 2, subfamily b, polypeptide 10	7 A3|7 6.5 cM			**−2.5753**	**0.0138**
10512999	NR_033139	**AI427809**	expressed sequence AI427809	4 B2			**−2.5806**	**0.0109**
10405063	NM_008760	**Ogn**	osteoglycin	13 A5			**−2.6878**	**0.0346**
10512949	NM_013454	**Abca1**	ATP-binding cassette, sub-family A (ABC1), member 1	4 A5-B3|4 23.1 cM			**−2.753**	**0.0273**
10366043	NM_026268	**Dusp6**	dual specificity phosphatase 6	10 C3			**−3.0369**	**0.0224**
10534493	NM_019577	**Ccl24**	chemokine (C-C motif) ligand 24	5 G1			**−4.9474**	**0.0072**
10463023	NM_001011707	**Cyp2c66**	cytochrome P450, family 2, subfamily c, polypeptide 66	19 C3			**−5.082**	**0.015**
